# Microglia depletion prevents lactation by inhibition of prolactin secretion

**DOI:** 10.1016/j.isci.2023.106264

**Published:** 2023-02-24

**Authors:** Vivien Csikós, Szilvia Oláh, Fanni Dóra, Nikolett Arrasz, Melinda Cservenák, Arpád Dobolyi

**Affiliations:** 1 Department of Physiology and Neurobiology, Eötvös Loránd University, Budapest, Hungary; 2 Laboratory of Neuromorphology, Department of Anatomy, Histology and Embryology, Semmelweis University, Budapest, Hungary

**Keywords:** Rodent behavior, Rodent physiology, Behavioral neuroscience, Endocrinology

## Abstract

Microglial cells were eliminated from the brain with sustained 3–4 weeks long inhibition of colony stimulating factor 1 receptor by Pexidartinib 3397 (PLX3397). The prepartum treated mice mothers did not feed their pups after parturition. The pups of mothers treated orally only in the postpartum period starting immediately after parturition showed reduced body weight by 15.5 ± 0.22 postnatal days as the treatment progressed without the mothers showing altered caring behaviors. The apparent weight gain of foster pups during a suckling bout was reduced in mother mice fed by PLX3397-containing diet and also in rat dams following sustained intracerebroventricular infusion of PLX3397 in a separate experiment suggesting that lactation was affected by the reduced number of microglia. Prolactin secretion and signaling were markedly reduced in PLX3397-treated mothers. The results suggest that microglial cells are required for prolactin secretion and lactation whereas maternal motivation may not be directly affected by microglia.

## Highlights

• Inhibition of colony-stimulating factor 1 receptor by PLX3397 depleted microglia
• Lactation was deficient following oral and intracerebroventricular PLX3397 injection
• Prolactin signaling in the brain was dramatically reduced after PLX3397 treatment
• Suckling-induced prolactin release was blocked in PLX3397 treated mothers

## Graphical abstract

**Figure:**
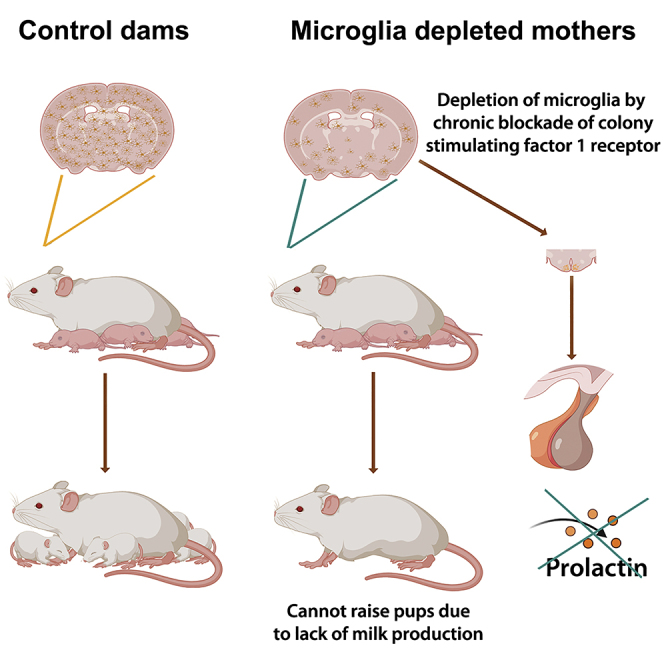

## Introduction

Microglia are tissue-resident macrophage-like innate immune cells of the central nervous system. They continuously examine the parenchyma of the brain, showing rapid activation in response to deleterious stimuli.^1^^,^^2^ Most studies on microglia focused on their role in pathophysiological brain processes.^3^^,^^4^ More recently, it was also established that microglia are multifunctional cells, which interact with neurons to contribute to neuroplasticity even under normal physiological functioning of the brain.^5^^,^^6^ In this study, in turn, we addressed the role of microglial cells in the process of maternal adaptation of the brain.

Becoming a mother involves major physiological, endocrinological, and behavioral changes that begin during pregnancy and continue after parturition. Maternal alterations of behavior represent the most profound non-pathological behavioral changes in the adult mammals. The accompanied adaptations of the maternal brain are important parts of mammalian reproduction. During pregnancy, placental lactogens, prolactin and steroid hormones prepare the brain to be ready for taking care of the offspring by parturition.^7^ These hormones are also crucial for mammogenesis and contribute to the formation of maternal behavior in the prepartum period.^8^ After parturition, stimuli from the pups maintain maternal motivation and caring behavior, such as nest building, licking, cleaning and retrieving the offspring to the nest.^9^^,^^10^ Although steroid levels are low in the postpartum period,^11^ suckling exerts its action on maternal behavior and lactation by direct neuronal pathways.^12^ The major brain site where both hormonal and neuronal inputs exert their effects on maternal behavior is the medial preoptic area where lesions, opto- and chemogenetic manipulations had major effects on maternal responses.^13^^,^^14^ The other prominent event taking place in the postpartum period is lactation, which is regulated by prolactin secreted from the lactotrophs in the anterior lobe of the pituitary gland^15^ to drive lactogenesis and galactopoiesis.^16^ The major signal transduction pathway of the prolactin receptor is via phosphorylated signal transducer activator, transcription 5 (pSTAT5).^17^^,^^18^ In mothers, the strongest physiological stimulus of prolactin release is the suckling stimulus exerted by the pups,^19^ which causes the mother’s serum prolactin level to rise several folds above baseline.^20^^,^^21^ In the absence of pups, prolactin secretion in mothers stops, and serum prolactin level becomes low.^22^ Suckling-induced prolactin secretion peaks 15–30 min after returning the pups to the mothers for suckling in the rat^23^ and the mice.^24^ Prolactin secretion from the pituitary is controlled by the inhibitory effect of dopamine released into the median eminence by dopaminergic neurons residing in the arcuate nucleus of the hypothalamus.^25^

Microglia mediate neuroplasticity, which is known to take place in the brain of pregnant and postpartum females related to behavioral changes^26^^,^^27^ and prolactin release.^28^ Therefore, we hypothesized that microglial cells play a role in central maternal adaptation as both lactation and behavioral alterations require long-term alterations in the neural function of the responsible neural circuitries. Furthermore, microglial activation increases in the pituitary gland of prolactinomas rats and microglial inflammasomes were identified as potential therapeutic targets.^29^ Recently, manipulation of microglia became available as Colony-stimulating factor 1 (CSF1)^30^ was shown to control microglial proliferation and differentiation through its transmembrane receptor, the CSF1 receptor (CSF1R).^31^ CSF1R is a receptor tyrosine kinase that belongs to the family of platelet-derived growth factor receptors.^32^ CSF1R-mediated signaling is required for the maintenance of microglial populations.^33^^,^^34^ Pexidartinib3397 (PLX3397) is one of the lead oral tyrosine kinase inhibitors of CSF1R.^35^ This drug crosses the blood brain barrier^36^ and inhibits CSF1R in microglia. Although CSF1R is involved in macrophage proliferation, differentiation, and survival, its blockade leads to the disappearance of microglia from the central nervous system^37^ without significant effects on peripheral immune cells.^38^ It was demonstrated that diet-based PLX3397 treatment is also effective in the depletion of microglia from the brain.^37^ In mice, oral PLX3397 treatment reduced the number of microglial cells by 14 but not 7 days after the beginning of treatment with the plateau of reduction in the number of microglial cells taking place by 21 days after treatment started.^39^ In rats, PLX3397 was also similarly effective in eliminating microglia cells from organotypic cultures^40^^,^^40^ as well as *in vivo* following oral PLX3397 treatment.^41^ Such depletion of microglia cells had negative consequences in stroke models^3^^,^^4^ without affecting normal behaviors of the animals.^42^

In the present study, we removed microglial cells from the brain by blocking the CSF1R with PLX3397. In the first experiment, PLX3397 was orally administered starting from the first day of pregnancy in mice. To eliminate the possibility that the embryonic development of the pups and the prepartum processes in the mothers are affected by the treatment, oral PLX3397 treatment started only following parturition in the second experiment. Because PLX9974 treatment fully effectively eliminates microglial cells from the brain only 21 days after treatment started,^39^ we utilized the fact that lactation can be maintained by adding foster pups to the mothers^43^^,^^44^ to examine the effect of sustained treatment on lactation. In the third experiment, PLX3397 was administered intracerebroventricularly into the lateral ventricle of dams starting in the postpartum period to eliminate potential peripheral effects of the drug. In this experiment, mother rats were used so that we provide a broader applicability of the observations across different species. Dams were investigated for their ability to feed the young, prolactin signaling and release. To assess the effect of the disappearance of microglia from the brain on behavioral changes, maternal behaviors were measured in all 3 experiments (Figure S1). Locomotor, anxiety- and depression-like behavioral tests were also performed to obtain information on the general condition of the dams. Finally, pups received PLX3397 to exclude the possibility that their reduced weight gain is mediated by a direct drug effect on them.

## Results

### Microglia depletion from the brain

**Figure 1:**
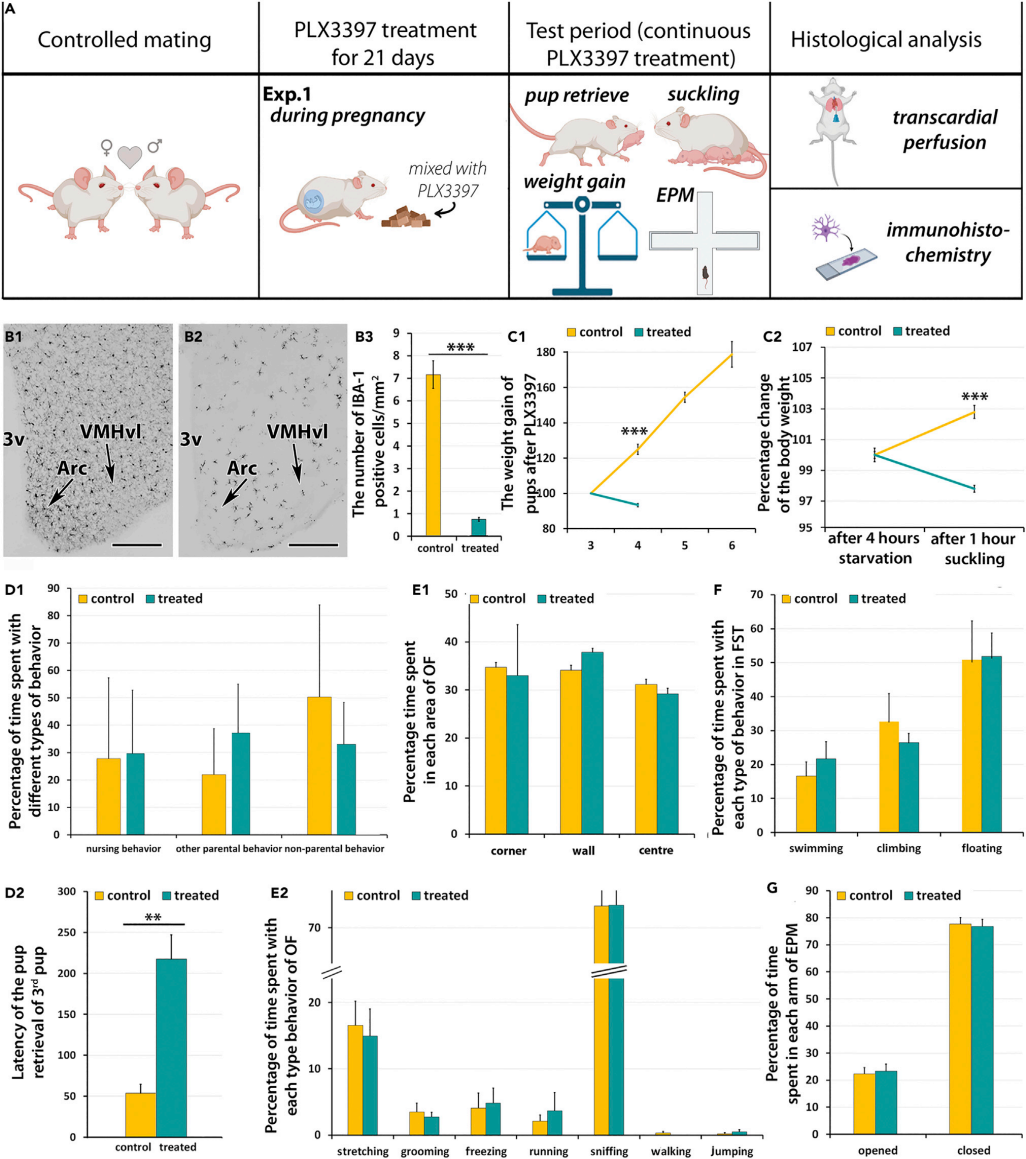
The effect of systemic PLX3394 treatment on the body weight of pups and prolactin signaling and behavior in mice whose treatment started during pregnancy (Experiment 1 - Exp. 1) (A) The timeline of the protocols. The mating of animals (n = 6 animals per group) was controlled for accuracy of the treatment. (B) Disappearance of Iba1-positive microglial cells in response to PLX3397-treatment. b1, Iba1-labeled coronal section of the mediobasal hypothalamus in control animals. Labeled cells are shown in the arcuate nucleus (Arc) and the ventromedial subdivision of the ventrolateral hypothalamic nucleus (VMHvl). b2, The same field of a coronal section as in b1 is shown in a PLX3397 treated animal demonstrating a reduced number of microglia cells. The scale bar is 300 μm. b3, The number of Iba1 labeled cells per 1 mm^2^ are shown in the Arc. The column representing the density of Iba1 labeled cells is yellow for the control animals and green for the treated group (n = 6 mothers per group). Statistical analysis was performed using two-way ANOVA test with the type of treatment (PLX3397 versus control) being one parameter and the brain regions being the repeated parameter followed by Sidek’s multiple comparison test. (C) The effect of PLX3397 treatment on the body weight of pups (n = 18 pups) in Exp. 1. The statistical analysis of weight gain was performed with two-way ANOVA with the treatment being the independent parameter and the body weights on consecutive days the repeated parameter. C1, The change of the body weight of pups in Exp. 1 in percentage of body weight at postpartum day 3 (test day 2, treated day 23). A large difference was detected the following day when the measurement ended for the treated mothers to save the life of pups. C2, The apparent body weight change of pups during a 1-h suckling bout after 4 h of maternal separation in Exp. 1. D1, The percentage of the time mothers (n = 6 mothers per group) spent with parental behavior in Exp. 1 when treated mothers received oral PLX3397 treatment during the pregnancy. There was no difference between the two groups (control group - yellow column, treated group – green column) in nursing, and other parental or non-parental behaviors. D2, The latency of retrieval of the third pup into the nest. There was a significant difference between control and microglia depleted groups in Exp. 1 as the treatment increased the latency of pup retrieval (n = 6 mothers per group). The statistical analysis was performed with unpaired t-test using the Welch’s correction because the F test indicated significant differences between the variances of the control and treated groups. E1, The effect of PLX3397 treatment on the behavior of mother (n = 6 per group) in an arena (open field test).e1, There was no difference between the groups in terms of the part of the arena they stayed. E2, Duration of behavioral elements exhibited by the two groups of mice did not differ, either. (F) The results of the forced swim test (FST). The animals did not show any difference in the time they spent with swimming, climbing and floating. (G) The results of the elevated plus maze (EPM) test. There was no difference in the time spent in the arms between the treated and control groups. Data are represented as mean ± SEM.

Brain depletion of microglial cells was successfully verified by immunolabeling with ionized calcium-binding adapter molecule 1 (Iba1), an established marker of microglia.^45^ In the arcuate nucleus of mice the number of microglia cells was reduced from 7.17 ± 0.62cell/mm^2^ in the control to 0.74 ± 0.09 cell/mm^2^ in the treated group when the oral PLX3397 treatment started during pregnancy (Exp. 1; n = 6 mothers per group, Figure 1B). The number of microglial cells decreased markedly following oral PLX3397 treatment in other brain areas, too (F(1.23) = 77.93, p < 0.001; Table S1). Figure S2 shows pictures of some of these Iba1-immunolabeled brain regions. The decrease in the density of Iba1-positive cells was similar when the mice received oral PLX3397 treatment after parturition in Exp. 2. The number of Iba1-immunolabeled cells in the arcuate nucleus was 7.05 ± 0.84 cell/mm^2^ in the control animals and 1.03 ± 0.18 cell/mm^2^ in the treated group. Comparing several brain areas, the amount of Iba1 decreased significantly (F(1.48) = 8.679, p < 0.01; n = 6 mothers per group). Following intracerebroventricular injections of low concentration of PLX3397 (0.5 mg/mL infused via osmotic minipump with a 0.25 μL/h injection rate) into the lateral ventricle of rats in Exp. 3, the number of microglial cells decreased in the arcuate nucleus from 15.20 ± 0.74 cell/mm^2^ in the control group to 12.46 ± 1.06 cell/mm^2^ in the treated group (p < 0.05, n = 10 treated and 9 control mothers, Figure S3) whereas a tendency of decrease in the number of microglial cells was found in other brain regions (Table S1).

### Experiment 1: Effect of microglia depletion during pregnancy and postpartum on pup weight gain, and maternal behaviors

#### Weight gain of pups in response to microglia depletion

The body weight of the pups on the third postpartum day (second test day, 23rd treatment day), when we first measured them, was an average of 1.36 ± 0.05 g in the control group (n = 18 pups of 6 mothers) whereas the pups of the PLX3397 treated group (n = 6 mothers) did not survive until that day in Exp. 1. The weight of each pup at postnatal day 3 was considered 100%. The change of the body weight increased to 124.92 ± 2.98% by the fourth postpartum day (third test day, 24th treatment day), 154.48 ± 2.81% by the fifth day (fourth test day, 25th treatment day), and 178.81 ± 7.31% by the sixth day (fifth test day, 26th treatment day) in the control group (n = 18 pups of 6 mothers). When foster pups were given to the treated mothers (3 pups per mother) on the third postnatal day (second test day, 23rd treatment day) to test if defects in mothers or pups were the reason for the lack of weight gain of pups, the body weight of the foster pups was decreased to 93.36 ± 0.98% of their initial body weight by the following day when the examination of the treated animals was terminated because of the weight loss of the pups (Figure 1c1; Table S2).

#### The apparent body weight gain of pups during a suckling bout

To find out if the decline in body weight in the treated group was a consequence of insufficient milk consumption because of a defect in mothers or pups, the apparent weight gain of foster pups (Table S3) during a 1-h long suckling was examined on the third postpartum day (second test day, 23rd treatment day) in Exp. 1. To enhance suckling of the pups, they underwent a 4-h separation from their mothers and then they were given to the experimental mothers as foster pups. The body weight of the pups at this time point was considered 100%. All pups started to suckle within 5 min of being given to the mothers. Their apparent body weight was measured after 1 h of suckling. Although the apparent body weight of pups given to control dams increased to 102.80 ± 0.87%, the apparent body weight of pups returned to mothers treated with PLX3397 during pregnancy decreased to 97.81 ± 0.72%. The difference in apparent body weight gain between the two groups of pups was highly significant (p < 0.0005; n = 18; Figure 1c2; Table S4).

#### Maternal behavioral changes

The dams (n = 6 mothers per group in Exp. 1) showed maternal care in both the control and the PLX3397 treated groups. Testing of the maternal behavior was performed on the second test day. The nursing behavior, other maternal care behaviors (pup sniffing, nest building, time spent in the nest) and non-parental behaviors (exploration, digging, freezing, self-grooming) were evaluated and no difference was found between the experimental groups (Figure 1d1). The p value was 0.8502 with two-way ANOVA-test in Exp. 1 (Figure 1d1). The results on maternal behaviors are summarized in Table S5.

Pup retrieval tests were performed in the first test day in Exp. 1 (second postpartum day, 22nd treatment day). There was a significant difference in pup retrieval time (in seconds) between the treated (217.53 ± 29.46) and control (53.87 ± 10.83) groups of mothers. The pvalue was 0.0017 with unpaired t-test using Welch’s correction indicating a difference between the treated and control groups (Figure 1d2).

#### Locomotor, anxiety- and depression-like behaviors

The mother mice who received PLX3397-treatment did not exhibit significant difference in the examined tests of locomotion, anxiety- and depression-like behaviors as compared to the controls determined with Student’s *t* tests. The results of the open field test are shown in Figure 1e1 and e2 when dams received the treatment during pregnancy (Exp. 1). The effect of treatment on the forced swim test is shown in Figure 1F. In the elevated plus maze test, there was no significant difference between the control and treated groups, either (Figure 1G; Table S6).

### Experiment 2: Effect of microglia depletion during lactation on pup weight gain, prolactin signaling and maternal behaviors

To eliminate possibilities that pups can be directly affected by the drug through the placenta and also that maternal adaptation during pregnancy, e.g., development of mamma were affected in Exp. 1, the administration of PLX3397 started after parturition in the second experiment.

#### Weight gain of pups in response to microglia depletion

**Figure 2:**
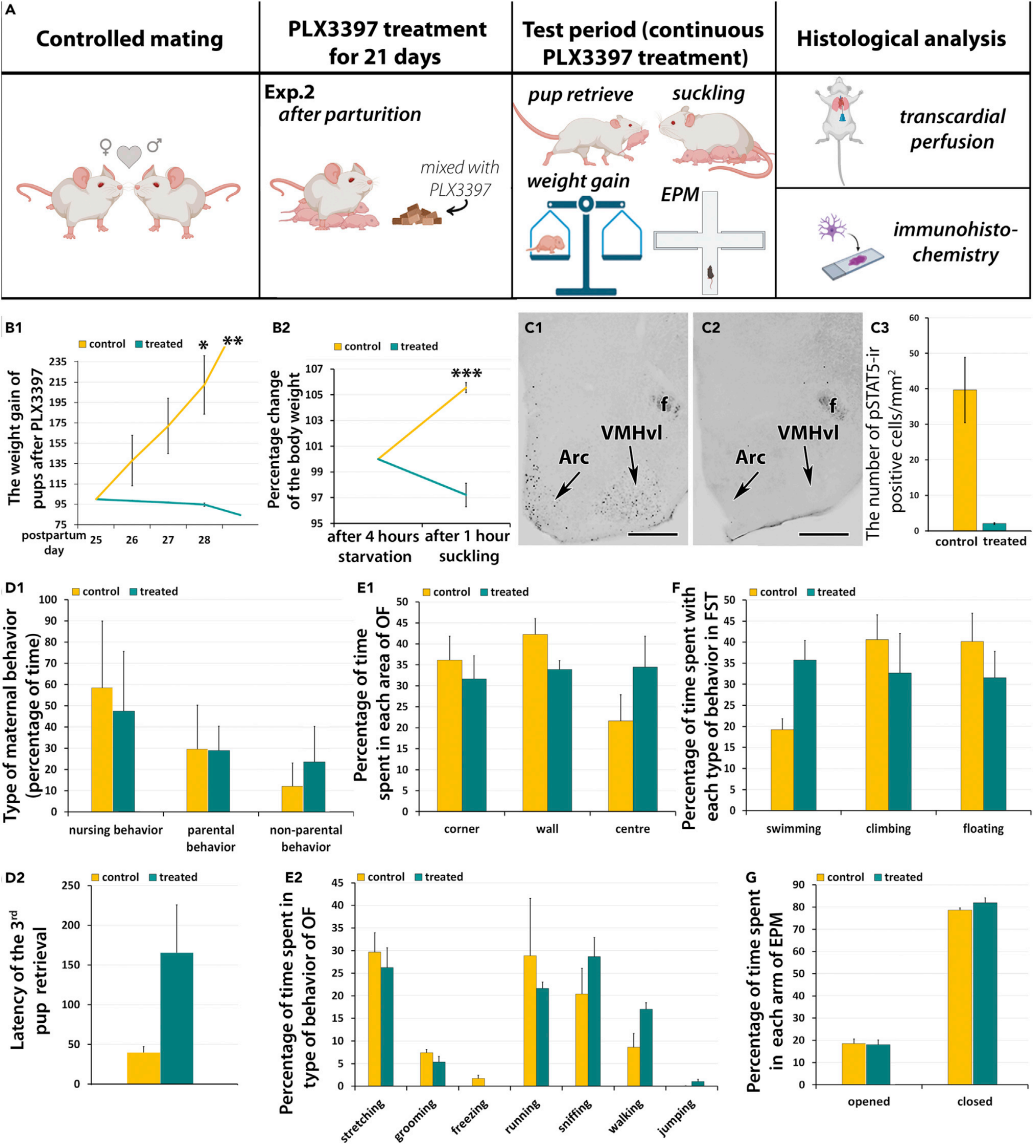
The effect of systemic PLX3394 treatment on the body weight of pups and prolactin signaling and behavior in mice whose treatment started during pregnancy (Experiment 2) (A) The timeline of the protocols. The mating of animals (n = 6 animals per group) was controlled for accuracy of the treatment. (B) The effect of PLX3397 treatment on the body weight of pups (3 pups/mother) in Exp. 2 (n = 6 mothers per group). The statistical analysis of weight gain was performed with two-way ANOVA with the treatment being the independent parameter and the body weights on consecutive days the repeated parameter. b1, The change of the body weight of foster pups given to mothers at their 21st postpartum day in Exp. 2. The body weights of the foster pups are expressed in percentage of their body weight at the 23rd postpartum day (second test day, 23rd treated day) of the mothers. The body weight of the pups was reduced 3 days later for the treated mothers. b2, The effect of the treatment on the body weight gain of pups during a 1 h long suckling bout following 4 h of maternal separation performed at the 25th postpartum day (fourth test day, 25th treated day) of the mothers using foster pups. The apparent weight gain of pups during suckling was highly significantly lower in the treated than the control group. (C) The effect of PLX3397 treatment on the number of prolactin sensitive cells in the mediobasal hypothalamus (n = 6 mothers per group). C1, pSTAT5-positive cells (black dots) are present in the Arc and the VMHvl of a control animal. C2, A brain section showing the same field as in e1 demonstrates that pSTAT5-positive neurons are not visible in the Arc and VMHvl of a PLX3397-treated animal. The scale bar is 300 μm. C3, Quantification of the density of pSTAT5-positive cells in the mediobasal hypothalamus. The columns represent the number of pSTAT5-positive cells in the control animals (yellow) on the left and the treated group (green) on the right. Additional abbreviations: 3V – third ventricle, f – fornix. ∗: p < 0.05; ∗∗∗: p < 0.001. D1, The effect of postpartum oral PLX3397 treatment on the maternal behavior of dams. The columns show the percentage of time what mothers spent with nursing, other maternal behaviors, or non-parental behaviors. D2, The effect of postpartum oral PLX3397 treatment on behavior of mice dams in the pup retrieval test. E1, The effect of PLX3397 treatment on the behavior of mother (n = 6 per group) in an arena (open field test). There was no difference between the groups in terms of the part of the arena they stayed. E2, Duration of behavioral elements exhibited by the 2 groups of mice did not differ, either. (F) The results of the forced swim test (FST). The animals did not show any difference in the time they spent with swimming, climbing and floating. (G) The results of the elevated plus maze (EPM) test. There was no difference in the time spent in the arms between the treated and control groups. Data are represented as mean ± SEM.

In Exp. 2, when PLX3397 treatment started immediately after parturition (n = 6 mothers per group), the body weight of the pups of the treated mothers started to decrease at 15.5 ± 0.22 postnatal days. At the day of reduced body weight, the pups were exchanged for foster pups. In addition, on the 17^th^ postpartum day, all the pups of both the treated and control groups were changed to 4–5 days old foster pups (3 pups per mother) to maintain lactation by suckling of the pups as the original pups already would start to eat dry food, and lactation would decrease and eventually cease. To investigate the effect of sufficiently long (over 21 days) of PLX3397 treatment, by the time the drug effect is maximal on the depletion of microglia,^37^^,^^39^ new 2–3 days old foster pups (3 pups per mother) were given to all animals on the 21st postpartum day (21st treatment day). The weight of the foster pups given to control mothers was 1.29 ± 0.04gat that time. The weight of pups given to treated dams was 1.70 ± 0.07 g. On the 22nd postpartum day (first test day, 22nd treatment day), the body weight of pups was 1.69 ± 0.20 g in the control and 1.87 ± 0.08 g in the treated group. The weight of the pups was measured daily thereafter for 3 days as presented in Table S2. Taking the body weight of the foster pups 100% on the first test day (22nd postpartum day, 22nd treatment day), in the control group, the percentage change of the body weight of the foster pups was increased to 137.90 ± 24.85%, 171.98 ± 27.09%, and 212.21 ± 28.73% by test days 2, 3, and 4, (postpartum days 23, 24 and 25; treatment days 23, 24, 25) respectively. In contrast, the body weight of the foster pups given to the treated mothers was 98.32 ± 0.74%, 96.83 ± 0.52%, and 94.67 ± 1.71% on the same days (Figure 2b1; Table S2), which represents a significant reduction of body weight as compared to the control group (F(1.16) = 32.69, p < 0.0001). The pvalue was 0.268 on the second test day (23rd postpartum day, 23rd treatment day), 0.0044 on the third test day (24th postpartum day, 24th treatment day) and 0.0035 on the fourth test day (25th postpartum day) (Figure 2b1; n = 18; Table S2).

#### The apparent body weight gain of pups during a suckling bout

Milk consumption during an acute suckling episode was measured on the first test day (22nd postpartum day, 22nd treatment day) of mothers treated with PLX3397 only during the postpartum period. The apparent body weight of foster pups was 105.57 ± 0.42% for control mothers and 97.21 ± 0.76% for treated mothers. It represents a significant reduction in apparent body weight gain of pups (p < 0.0001; Figure 2b2; Table S4).

#### The number of prolactin signaling neurons following suckling

Because prolactin is the major hormone responsible for milk production, we aimed to test if prolactin action is present in the mothers. The signal transduction protein of prolactin receptor, pSTAT5 was used for this purpose. Following suckling, pSTAT5-immunolabeled neurons appeared in several different brain regions including the arcuate nucleus (Arc) and the ventrolateral subdivision of the ventromedial hypothalamic nucleus (VMHvl) (Figure 2C) when investigated in Exp. 2. There was a significant difference in the number of pSTAT5-immunolabeled cells between control (Figure 2c1) and PLX3397 treated dams (Figure 2c2). The number of pSTAT5-ir cells in the arcuate nucleus of control mothers was 39.67 ± 9.20/mm^2^ whereas in dams treated with PLX3397 it was 13.87 ± 3.89/mm^2^ (Figure 2c3), a significant reduction in response to treatment (p < 0.005, n = 6 mothers per group) determined with unpaired t-test.

#### Maternal behaviors

The dams (n = 6 mothers per group in Exp. 2) showed maternal care in both the control and the PLX3397 treated groups. Testing of the maternal behavior was performed on the second test day. The nursing behavior, other maternal care behaviors (pup sniffing, nest building, time spent in the nest) and non-parental behaviors (exploration, digging, freezing, self-grooming) were evaluated and no difference was found between the experimental groups (Figure 2d1). The p value was 0.348 with two-way ANOVA-test in the postpartum treated animals in Exp. 2 (Figure 2d1).

Pup retrieval tests (n = 6 mothers per group) were performed in the first test day in Exp. 2 (22nd postpartum day, 23rd treatment day). The pvalue was 0.0899 between the treated (165.87 ± 60.21) and control (39.43 ± 7.41) groups of mothers using unpaired t-test with Welch’s correction showing a trend toward reduction in response to the treatment (Figure 2d2).

#### Locomotor, anxiety- and depression-like behaviors

In Exp. 2 when the mothers were treated only postpartum, there were no significant differences between the groups in the any of the behavioral tests of locomotion, anxiety- and depression-like behaviors (Figures 2E–2G; Table S6).

### Experiment 3: Effect of microglia depletion by postpartum intracerebroventricular PLX3397 treatment of lactating rats on pup weight gain, prolactin signaling, prolactin secretion, and maternal behaviors

#### The effect of intracerebroventricular PLX3397 treatment on the body weight of pups

**Figure 3:**
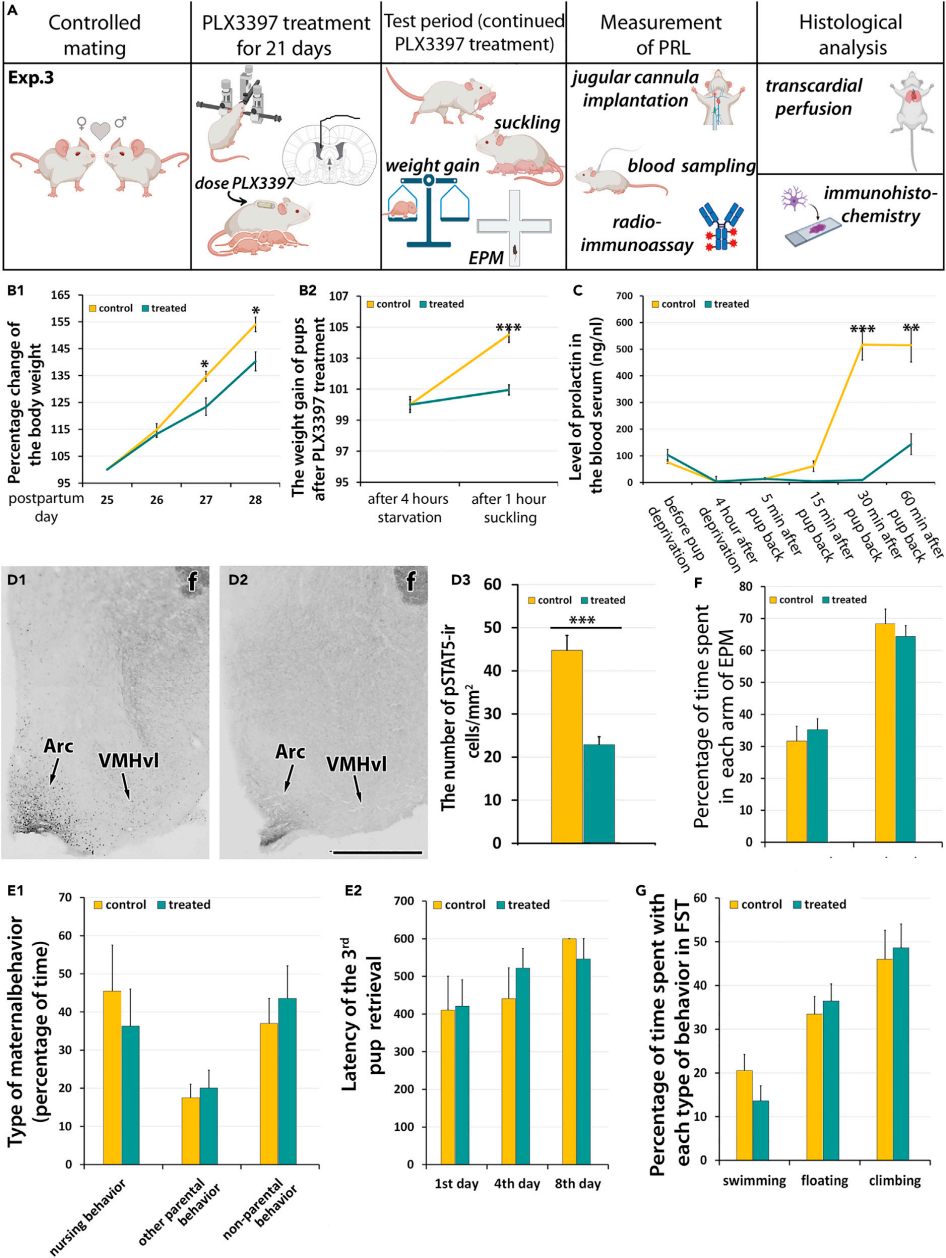
The effect of intracerebroventricular PLX3394 treatment of postpartum mothers on the body weight of pups and prolactin secretion and behavior in rats (Experiment 3) (A) Illustration of the third experiment (Exp. 3), in which rat mothers (n = 10 treated and 9 control mothers) received PLX3397 treatment only in the postpartum period directly to the lateral ventricle via osmotic minipumps. (B) The effect of PLX3397 treatment on the weight of litters (10 pups per litter; n = 9 litters for control and 10 for treated mothers). The statistical analysis of weight gain was performed with two-way ANOVA with the treatment being the independent parameter and the body weights on consecutive days the repeated parameter. B1, The change of the body weight of foster pups given to mothers at their 23rd postpartum day in Exp. 3. The body weight of the foster pups is expressed in percentage of their body weight at the 25^th^postpartum day (first test day, 23^rd^ treated day) of the mothers. The body weight of the pups was reduced 2 and 3 days later for the treated mothers. B2, The weight gain of pups during a 1-h long suckling bout. Although pups of treated mothers gained some weight during suckling, the weight gain was significantly higher in the control group (p < 0.001). (C) The effect of PLX3397 treatment on suckling-induced serum prolactin levels. The level of prolactin was equally low in the 2 groups after 4 h of pup separation. In turn, significant difference was found 30 and 60 min after the pups were given back to the mothers (p < 0.001, n = 10 treated and 9 control mothers) as the serum prolactin level was dramatically increased in the control group whereas only a small and late increase was detected in the treated mothers. The statistical analysis was carried out with two-way ANOVA followed by Sidak’s multiple comparison test with the type of treatment (PLX3397 versus control) being one parameter and time points of prolactin level measurements being the repeated parameter. ∗: p < 0.05; ∗∗: p < 0.01, ∗∗∗: p < 0.001. d1, pSTAT5-ir cells (black dots) are present in the Arc and VMHvl nuclei in a control rat. D2, The brain section represents the corresponding field as in d1 in a PLX3397-treated mother. It is demonstrated that the treatment causes a reduced number of pSTAT5-positive neurons. The scale bar is 300 μm. D3, Quantification of the number of pSTAT5-ir cells in the mediobasal hypothalamus. The columns represent the density of pSTAT5-ir cells in the control animals (yellow) on the left and the treated group (green) on the right. f – fornix. The statistical analysis to compare the density of cells in the mediobasal hypothalamus between control and PLX3397 treated groups was performed with unpaired t-test. ∗∗∗: p < 0.001. E1, The effect of postpartum oral PLX3397 treatment on the maternal behavior of dams. The columns show the percentage of time what mothers spent with nursing, other maternal behaviors, or non-parental behaviors. E2, The effect of postpartum oral PLX3397 treatment on behavior of mice dams in the pup retrieval test. (F) The results of the elevated plus maze (EPM) test. There was no difference in the time spent in the arms between the treated and control groups. (G) The results of the forced swim test (FST). The animals did not show any difference in the time they spent with swimming, climbing and floating. Data are represented as mean ± SEM.

To determine the effects of the central actions of PLX3397, infusion of PLX3397 into the lateral ventricle of mother rats (n = 10 treated and 9 control mothers) started on the second postpartum day with the implantation of osmotic minipumps. On the 17th postpartum day, foster pups (10 pups per mother) were given to the mothers to maintain their lactation and maternal care behavior. On the 23rd postpartum day, 3–5 days old foster pups (10 pups per mother) were given to the dams. On the 25th postpartum day (first test day, 23rd treated day), the body weight of foster pups given to control mothers was 7.93 ± 0.97 g whereas the body weight of foster pups given to treated mothers was 9.63 ± 1.49 g. The weight of pups was measured the subsequent three days, too. The body weight of foster pups on the first test day (25th postpartum day, 23rd treated day) was considered 100%. The weight of pups given to control mothers increased as expected. Compared to the values measured on the first test day (25th postpartum day, 23rd treated day), it was 114.84 ± 2.25% the next day, and 134.73 ± 1.86% and 154.08 ± 2.71% 2 and 3 days later, respectively. In contrast, the body weight of pups given to treated mothers was only 113.20 ± 1.26% on the following day, 123.40 ± 3.24% and 140.27 ± 3.55% 2 and 3 days later, respectively. The difference in the body weight between the treated and control groups was significant (F(1.68) = 4.4226, p < 0.05, n = 10 treated and 9 control mothers). Using Sidak’s post-hoc test, the pvalue was 0.268 on the second test day (26th postpartum day, 24th treatment day), 0.0044 on the third test day (27th postpartum day, 25th treatment day) and 0.0035 on the fourth test day (28th postpartum day, 26th treatment day; Figure 3b1; Table S2).

#### The effect of intracerebroventricular PLX3397 treatment on the suckling of pups

During a 1-h long suckling bout performed on the first test day (25th postpartum day, 23rd treatment day), the percentage change of the apparent body weight was 104.51 ± 2.48% in control and 100.95 ± 1.04% in the treated group, which represents a significant decrease in the treated group (p < 0.001, n = 10 treated and 9 control mothers; Figure 3b2; Table S3).

#### Suckling-induced serum prolactin level

Prolactin level in the blood serum was measured in Exp. 3 when rats (n = 10 treated and 9 control mothers) received the PLX3397-treatment into their lateral ventricle via osmotic minipump. The blood sampling through the jugular vein took place before pup deprivation, 4 h later, at the end of pup deprivation, and 5, 15, 30, 60 min after returning the pups. The prolactin level in control mothers was 75.79 ± 4.39 ng/nL before the deprivation. After 4 h of deprivation, the prolactin level was decreased to 3.73 ± 18.79 ng/nL 5 min after returning the pups, the level of prolactin in control animals was 14.12 ± 2.31 ng/nL. In turn, 15 min after giving back the pups, 61.43 ± 19.40 ng/nL prolactin was measured in the serum. 30 min after returning the pups, the serum prolactin level in control mothers was 516.91 ± 58.04 ng/nL, whereas 514.17 ± 63.13 ng/nL prolactin was measured at the 60 min time point (Figure 3C).

The baseline value of prolactin serum level in treated mothers was 103.78 ± 38.95 ng/nL. After pup deprivation, the serum prolactin level was reduced to 4.10 ± 0.50 ng/nL 5 min after returning the pups, the value of prolactin level was 14.19 ± 3.86 ng/nL 15 min after returning the pups, the prolactin level in the treated mothers was 4.36 ± 0.49 ng/nL. In turn, 8.90 ± 1.22 ng/nL was measured 30 min after returning the pups. At 60 min, the prolactin level was 143.77 ± 38.82 ng/nL (Figure 3C). The difference was significant between the time points and also the treatments. The reduction in suckling-induced serum prolactin level in response to PLX3397 treatment was significant as compared to the control mothers (F(1.83) = 16.44, p < 0.0001 with two-way ANOVA-test, n = 10 treated and 9 control mothers). The Sidak’s multiple comparison post-hoc test was significant at 30 min (p < 0.001) and at 60 min (p < 0.0001).

#### The number of neurons showing prolactin-induced signal transduction

To test prolactin signaling in rats, pSTAT5 was immunolabeled in the mothers whose suckling-induced prolactin level had been examined. pSTAT5-immunolabeled neurons appeared in several different brain regions including the arcuate nucleus (Arc) and the ventrolateral subdivision of the ventromedial hypothalamic nucleus (VMHvl) (Figure 3D). There was a significant difference in the number of pSTAT5-immunolabeled cells between control (Figure 3d1) and PLX3397 treated dams (Figure 3d2). The number of pSTAT5-ir cells in the ventrobasal hypothalamus was 46.63 ± 3.67/mm^2^ in control mother rats and 22.84 ± 1.80/mm^2^ in intracerebroventricularly treated rat dams (Figure 3c3), a significant reduction in response to treatment (p < 0.005) determined with unpaired t-test.

#### Behavioral effects

Testing of the maternal behavior was performed on the second test day in Exp. 3, too (n = 10 treated and 9 control mothers). The nursing behavior, other maternal care behaviors (pup sniffing, nest building, time spent in the nest) and non-parental behaviors (exploration, digging, freezing, self-grooming) were evaluated and no difference was found between the experimental groups. The p value was 0.343 when the injection was into the lateral ventricle in Exp. 3 (Figure 3e1).

The latency of pup retrieval for the intracerebroventricularly treated dams was determined on first (410.26 ± 90.19 versus 420.58 ± 70.40), fourth (440.75 ± 81.82 vs. 521.74 ± 52.72) and eighth test days (600.00 ± 0.00 versus 546.42 ± 53.58) in control and treated groups, respectively. The difference between the treatments was examined with two-way repeated measure ANOVA (p = 0.6939; F(1. 17) = 0.1603), which did not indicate an effect of the PLX3397 treatment (Figure 3e2).

In addition, there were also no significant differences between the groups in the any of the behavioral tests of locomotion, anxiety- and depression-like behaviors (Figures 3F and 3G; Table S6).

### The effect of PLX3397 injection into the pups on their body weight and suckling

To test if PLX3397 can directly affect the suckling pups, we measured their body weight following injection of the drug into the pups. Intraperitoneal injection of PLX3397 (100 mg/kg) into the pups had no effect of the body weight increase of pups. The weight of the control group was 6.14 ± 0.11g at the time point of vehicle injection and 6.24 ± 0.14 g the following day. The treated group had an initial body weight of 5.78 ± 0.08 g immediately before PLX3397 injection and 5.86 ± 0.07 g a day later.

**Figure 4:**
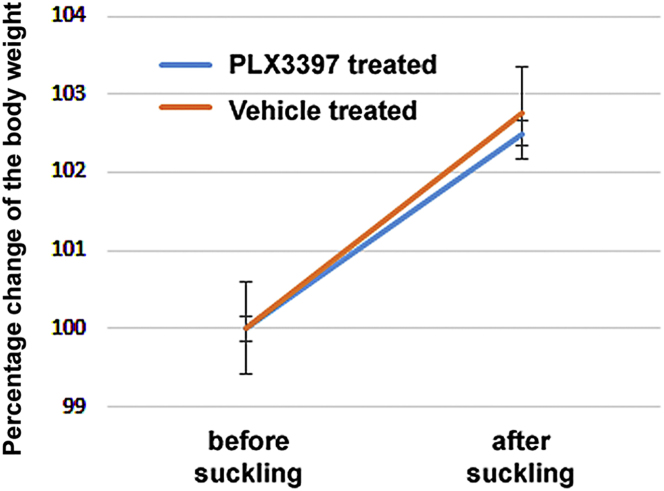
The effect of PLX3398 treatment of the pups on the body weight during a suckling bout Vehicle and PLX3397 treated pups gained similar amounts of weight during a 1-h long suckling bout. Data are represented as mean ± SEM.

The following day, the pups were removed from the mothers for 4 h, and received another dose of PLX3397 1 h before being returned to their mothers. The apparent body weight gain of treated pups during a 1-h-long suckling bout did not differ from that of the vehicle injected group (Figure 4). The weight gain of the control group was 102.76 ± 0.59% whereas the weight gain of the treated group was 102.50 ± 0.16% when considering their body weight 100% immediately before returning them to their mothers.

## Discussion

Microglia sense changes in the integrity of the central nervous system and respond to its challenges. Over the past decade, the established roles of microglia has been extended to non-immunological functions, such as synaptic remodeling. During motherhood, a number of adaptations including endocrinological changes take place in the brain resulting in lactation,^46^ which is primarily driven by prolactin, a hormone with a cytokine receptor.^47^ Therefore, the identified specific role of microglial cells in the regulation of prolactin secretion is unique but not unexpected.

Microglia cells can be eliminated from the brain by inhibiting the CSF1R^48^ with sustained PLX3397 treatment.^37^^,^^49^ The highly significant decrease in the microglia content of the brain of dams, similar to previous description of PLX3397 effects in male mice,^37^^,^^49^ demonstrated the effectiveness of the treatment during pregnancy and the postpartum period, too. A major finding of the study is that although the behavior of the mother animals did not change substantially toward the pups, the survival of the offspring was markedly affected. In the first experiment, the treatment, requiring 21 days to fully exert the effect of PLX3397 on the number of microglial cells^37^^,^^39^ started during pregnancy. To eliminate the possibilities that the pups are directly affected by the drug during pregnancy or that the drug exerts its effects on the mothers by pregnancy-related processes, the experiment was repeated so that the treatment started only after parturition. The weight of the pups of the postpartum treated mothers became significantly reduced by the 16th postpartum day without alteration in the caring behavior of the mothers in this experiment suggesting that lactation may be specifically affected and also that postpartum effects of PLX3397 are sufficient to inhibit lactation. At this point, younger foster pups were given to the mothers to maintain caring behavior and lactation until the treatment attained its full potency to reduce the density of microglial cells. Because we cannot exclude the possibility that PLX3397 reached the pups of the treated mothers via the circulation during pregnancy or via the milk during lactation, further experiments were performed with new foster pups. Even though the behavior of the treated mothers toward these pups was not different than in the control group, the weight gain of the foster pups was markedly reduced. In addition, the apparent weight gains of the pups during a suckling bout, caused by the milk the pups consume, was also markedly reduced suggesting that sufficient amount of milk was not produced by the treated animals. To rule out the possibility that PLX3397 reaching the foster pups via the consumed milk affects the suckling ability of the pups, PLX3397 was directly injected to foster pups, which had no effect of their apparent weight gain during a suckling bout implying that defects in the mother lead to reduced milk production.

The finding that the weight of pups of postpartum treated mothers became reduced by 15.50 ± 0.22 days after the beginning of the treatment demonstrated that this is the time required for the treatment to be effective. It is in line with previous studies using the same concentration of oral PLX3397, which demonstrated reduced density of microglial cells in the brain by 14 but not 7 days following the beginning of treatment.^39^ Such a long time required for the action of the treatment suggests that it is not an acute but a chronic action of the drug, which evokes the effects. This chronic effect is likely the reduction in the number of microglial cells^37^^,^^50^ although other unrelated mechanisms based on the kinase inhibitory action of PLX3397 cannot be fully excluded.

To address if central effects of PLX3397 caused the cessation of milk production, PLX3397 was chronically administered directly into the lateral ventricle of rat dams by osmotic minipumps. The amount of PLX3397 reaching the brain in this experiment was less than with oral treatment because of the low concentration of the intracerebroventricularly infused drug limited by its low water solubility. Although the mice consumed 6.92 ± 1.56 g of food daily, through which they received 2.00 ± 0.45 mg of PLX3397, the amount of drug administered daily into the ventricle of rats was 3 μg calculated by the 0.25 μL/h injection rate and the 0.5 mg/mL infused PLX3397 concentration. This low level of PLX3397, compatible with the 1 μM concentration of PLX3397 effective to eliminate microglia *in vitro*,^40^ was sufficient to partially deplete microglia from the brain and also to significantly reduce the weight gain of foster pups during a 1 h suckling bout suggesting central but not peripheral site of action of the drug even though PLX3397 can also inhibit macrophages. The alternative possibility that mice and rats are differently affected by PLX3397 is not likely based on previous data.^39^^,^^41^ The less than maximal effect on the weight loss of pups in this experiment suggests that greater microglia depletion is needed to elicit the full effect on lactation.

Lactation is driven by prolactin, a pituitary hormone contributing to milk production by acting on the mammary gland. Moreover, prolactin is actually required for milk production as lactation terminates in the absence of prolactin or prolactin receptor.^51^ In addition, prolactin also affects some neurons to inhibit its own secretion and also to promote maternal behaviors. The major signal transduction pathway of the prolactin receptor is via Janus kinase 3 (Jak3),^52^ whose product is pSTAT5, which is not present in brain when prolactin levels are low but can be immunohistochemically detected in response to extracellular prolactin administration^17^^,^^18^ or suckling.^12^ In our experiments, pSTAT5 indeed appeared following suckling in the control animals but only in reduced number of cells, following PLX3397 treatment. The reduction was present in orally treated mice as well as in intracerebroventrically treated rats. The degree of reduction was somewhat less in the rat, which is likely because of the late perfusion time necessary for measuring prolactin 60 min after returning the pups. In fact, we already measured some increase in serum prolactin level at this time point. The reduction in pSTAT5-ir cell density was also present in the arcuate nucleus, the brain area where pSTAT5 has the highest density and intensity (to serve as the feedback of prolactin to the dopaminergic neurons^53^). The reduced pSTAT5 may result from the low prolactin level. An alternative possibility that PLX3397 affects prolactin sensitive neurons by some way, which prevents them from forming pSTAT5 despite the presence of prolactin, is not very likely. In the intracerebroventricularly treated rats, we directly measured suckling-induced serum prolactin levels which were significantly reduced by PLX3397 treatment. Because PLX3397 level in this experiment was very low, the blockade was not complete, and a small amount of prolactin was secreted in response to suckling even in the treated group. Alternatively, species difference is also possible because the release of prolactin in male mice^28^^,^^54^ as well as prolactin release during estrous cycle and the prepartum period shows differences between mice and rats^55^^,^^56^ albeit the suckling-induced prolactin release has similar patterns in the two species.^24^^,^^57^ Nevertheless, the body weight of pups following intracerebroventricular treatment was reduced, but in contrast to the oral treatments, the pups could survive. Prolactin release from the lactotroph cells of the anterior lobe of the pituitary is mediated by the inhibitory action of dopaminergic neurons located in the arcuate nucleus.^25^^,^^58^^,^^59^^,^^60^ Therefore, it is plausible that these dopaminergic neurons are affected by the lack of sufficiently high density of microglial cells. Microglial cells could affect spines of dopaminergic cells or change their structural connectivity as both of these events were shown to alter their effect on prolactin release.^56^ In fact, the density of somatic spines is altered during lactation to a higher extent than at any other reproductive stage.^55^^,^^61^ Nevertheless, it cannot be excluded that PLX3397 treatment affects prolactin secretion by some other, indirect mechanism including direct action on the pituitary lactotrophs.

The effect of microglial cells is likely specific to neurons controlling lactation because behavioral actions of postpartum drug treatment were not found. It is not surprising because maternal motivation and behavior are largely considered to be hormone-independent in established lactation.^62^ The relatively small alterations in the behavior of prepartum treated mothers suggest that steroid hormones can create maternal motivation but a role of placental lactogens in contribution to establish maternal behavior is also plausible. In turn, postpartum PLX3397 treatment did not significantly affect maternal motivation and behavior. The lack of effect of PLX3397 on maternal behavior is similar to the lack of its effect on other behaviors reported previously^37^^,^^49^^,^^63^ suggesting that most behavioral output of the brain is not directly affected by microglial cells under normal circumstances. Following injury, the importance of microglia becomes apparent.^4^ For the release of prolactin, the situation is different because a physiological phenomenon is affected by the lack of microglial cells suggesting that microglial cells are involved in the control of prolactin secretion. Because the function of mediobasal dopaminergic neurons is known to be profoundly altered during lactation,^19^^,^^64^ we hypothesize that microglia contribute to their adaptation to lactation via neuroplastic mechanisms microglia are known to be involved.^27^^,^^65^ An intriguing further research based on these results is the investigation of microglia in the brain in pregnancy and lactation. It could even represent a negative feedback mechanism because prolactin can inhibit microglial function.^66^

### Limitation of the study

A *limitation of the study* is that the presented data are only correlative. Consequently, the mechanisms how the absence of microglia affects prolactin secretion have not been revealed. It is not even certain that microglia affects tuberoinfundibular dopaminergic neurons or exert their actions on prolactin secretion via other routes. Therefore, additional research will be needed to reveal the underlying mechanisms of microglia action.

### Conclusion

In conclusion, both oral and central administration of PLX3397 reduced microglia in the brain of dams and decreased the weight gain of the suckled pups whereas maternal behavior was not changed by postpartum drug treatment. These findings suggest a specific deficit in lactation. The absence of prolactin-induced pSTAT5 in the brains of mothers and reduced suckling-induced serum prolactin levels in the treated mothers suggest the inhibition of prolactin secretion.^23^ Thus, microglial cells may be necessary for prolactin release responsible for milk production, for the formation of maternal motivation via effect on prolactin release but not for the maintenance of maternal behaviors. In addition, the results also identify Colony-stimulating factor 1 in microglial cells as potential therapeutic targets in prolactinomas.

## STAR★Methods

### Key resources table

**Table:** 

REAGENT or RESOURCE	SOURCE	IDENTIFIER
**Antibodies**		

Rabbit Anti-Iba1 Antibody	FUJIFILM Wako Shibayagi	Cat. # 27030; RRID: AB_2314667
Rabbit Anti-pSTAT5 Antibody	Abcam	Cat. #: ab13593; RRID: AB_2239993
Biotin-SP (long spacer) AffiniPure Donkey Anti-Rabbit IgY (IgG)	Jackson ImmunoResearch	Cat. #: 711-065-152; RRID: AB_2340593

**Experimental models: Organisms/strains**		

Rat: wild type	Charles River Laboratories	Wistar
Mouse: wild type	The Jackson Laboratory	C57BL/6J

**Software and algorithms**		

ImageJ	Image Processing with ImageJ (Abràmoff et al., 2004)^67^	https://imagej.nih.gov/ij
Solomon Coder	Solomon Coder (Version Beta: 17.03.22) (Péter, 2016)^68^	https://solomon.andraspeter.com
SMART Video Tracking software	Panlab Harvard Apparatus	https://www.panlab.com/en/products/smart-video-tracking-software-panlab
GraphPad Prism	GraphPad Software, LLC	version 9.0.0., released 2020

**Chemicals, peptides, and recombinant proteins**		

Pexidartinib (PLX3397)	MedChemExpress, USA	Cat. # HY-16749
VECTASTAIN® Elite ABC-HRP Kit, Peroxidase	Vector Laboratories, Burlingame, CA, USA	Cat. # PK-6100
DAB Substrate Kit, Peroxidase (HRP), with Nickel, (3,3'-diaminobenzidine)	Vector Laboratories, Burlingame, CA, USA	Cat. # SK-4100
DePeX mounting medium	Sigma Aldrich	Cat. # 06522

### Resource availability

#### Lead contact

Further information and requests for resources and reagents should be directed to and will be fulfilled by the lead contact, Arpad Dobolyi (dobolyi.arpad@ttk.elte.hu).

#### Materials availability

This study did not generate new unique reagents.

### Experimental model and subject details

#### Animals

The Workplace Animal Welfare Committee of the National Scientific Ethical Committee on Animal Experimentation at Eötvös Loránd University, Budapest, approved this study (PE/EA/568–7/2020). The procedures involving rats and mice were carried out in accordance with the Hungarian Act of Animal Care and Experimentation and with the directive 2010/63/EU of the European Parliament and of the Council 22 September 2010 on the protection of animals used for scientific purposes.

A total of 24 female wild type, 2–4 monthsold C57BL/6J mice (n = 6 in both groups in Exp. 1 and 2) and 20 female Wistar rats were used in the study. One rat in the control group did not survive the implantation of osmotic minipump. Therefore, the control group in Exp. 3 consisted of only 9 while the treated group 10 rats. Mice were used for oral drug treatment while rats were used for intracerebroventricular drug treatment. For mating, 2 females and a male mouse/rat were kept together for 1 week. Then, pregnant females and mothers were housed individually until the end of the experiment in a separate room dedicated to the experiment. Experiments with mother mice were performed with 3 pups size litters while experiments with mother rats were performed with 10 pups size litter. The litter sizes were chosen because C57BL/6 strain of mice deliver 6.6 pups on average^69^ but litter number varies, which is in agreement with our experience. In turn, Wistar rats deliver 12-18 pups.^43^ Instead of the average number of pups per litter, we used 3 and 10 mice and rats, respectively, so that we do not have to exclude many mothers because of insufficient number of pups. Importantly, the onset in the decline of lactation is not related to litter size either in mice^44^ or in rat.^70^

All animals were kept in standard laboratory conditions; the temperature was kept constant (23 ± 1 °C) in 50–60% humidity, with 12-h light–dark cycle (lights on at 6:00 a.m). The animals were supplied with food (described below) (Ssniff, Soest, Germany) and drinking water *ad libitum*. All experiments were performed in the first phase of the light phase under controlled light (60 LUX) and temperature (23 ± 1 °C) conditions. Experimental animals were sacrificed by transcardial perfusion described below for histological examinations. Pups of the experimental animals were sacrificed by cervical dislocation followed by urethane anesthesia. Foster pups were weaned or returned to their mothers.

### Method details

#### Pexidartinib 3397 treatment

Pexidartinib 3397 (PLX3397) was purchased from MedChemExpress (USA, product number: HY-16749). It was mixed into the food of animals (PS R/M-H + 0.29 g/kg PLX3397; Ssniff) to reach a concentration of 290 mg/kg. Control animals received the same food without PLX3397 (SM R/M-H Placebo; Ssniff). Animals in the treated group were fed exclusively with the PLX3397-containing food throughout the experiments while control animals were fed with food of the same composition except for PLX3397.

Mice in the first experiment (Exp. 1, n = 6 mothers per group) received the treatment during pregnancy starting from the mating to the end of the experiments, which continued in the postpartum period. The treatment of the mice mothers in the second experiment (Exp. 2, n = 6 mothers per group) started immediately after parturition (Figure 1A). In the third experiment, mother rats (n = 10 mothers in the treated and n = 9 in the control group) received PLX3397 (0.5 mg/ml) infused via osmotic minipump with a 0.25 μl/h injection rate directly into their lateral ventricle starting on the 2^nd^ postpartum day as detailed below in STAR Methods. Treated mothers (just like the controls) did not show any observable sign of abnormality during the treatment in any of the experiments.

#### Histological analysis

##### Tissue collection for immunolabeling

Animals were sacrificed with urethane and transcardially perfused first with saline (30 ml for mice and 100 ml for rats) and then with 4% paraformaldehyde dissolved in 0.1 M phosphate buffer (PB; pH = 7.4; 100 ml for mice and 300 ml for rats). Brains were removed, postfixed in 4% paraformaldehyde for a day, and then transferred to PB containing 20% sucrose for a day for cryoprotection. Serial coronal sections were cut in 40 μm thickness with cryostat (Leica CM1520). Sections were collected in PB in 3 parallel series for mice and 5 series for rats used separately for individual staining. The sections were stored in PB containing 0.05% sodium azide at 4 °C until immunohistochemical procedures started.

#### IBA1 immunolabeling

The EF calcium-binding protein, Iba1, derived from the gene*iba1* (ionized calcium binding adapter molecule 1) is a protein expressed only in microglia in the brain.^71^ In mice, every third, while in rats, every fifth 40-μm-thick free-floating brain section of PLX3397-treated and control animals was processed for Iba1 immunohistochemistry. Sections from each experiment were pre-treated in PB containing 0.3% hydrogen peroxide (Sigma, St. Louis, Missouri, United States, Cat#: H1009) for 15 min for quenching of endogenous peroxidase activity. Then, the sections were incubated in PB containing 0.5% Triton X-100 (Sigma, Cat#: X100) and 3% bovine serum albumin (BSA; Sigma, Cat#: A3294) for 1 h. Sections were then incubated in rabbit anti-Iba1 antibody as a marker of microglial cells (1:1200; FUJIFILM Wako Shibayagi, Cat# 27030, RRID:AB_2314667) at room temperature for a night. Following the primary antibody, the sections were incubated in biotin-conjugated anti-rabbit IgG secondary antibody (1:800; Jackson ImmunoResearch, Cat#: 711-065-152) for 1 h and then in the avidin–biotin–peroxidase complex (ABC; 1:500; Vector Laboratories) for 1 h. The labeling was visualized by nickel-2’-diaminobenzidine (Ni-DAB) peroxidase technique. Sections were covered with Depex mounting medium (Sigma, Cat#:06522) after drying.

#### pSTAT5 immunolabeling

Every third 40-μm-thick free-floating brain section of PLX3397-treated and control animals in Exp. 2 was processed for pSTAT5 immunohistochemistry as described previously.^12^^,^^72^ Briefly, sections underwent an antigene retrieval treatment in Tris buffer (pH = 9.0) at 90°C for 10 min. Then, sections were treated with hydrogen peroxide and BSA as described above. Subsequently, sections were incubated in rabbit anti-pSTAT5 antiserum (1:100, Abcam, Cat#: ab13593, RRID: AB_2239993) at room temperature for 2 nights. Following the primary antibody, sections were incubated in biotin-conjugated anti-rabbit IgG secondary antibody (1:800; Jackson ImmunoResearch, Cat#: 711-065-152) for 1 h. Then, ABC reagent (Vector Laboratories) was used followed by Ni-DAB visualization as described above.

#### Analysis and quantification of immunolabeled cells

Brain areas containing Iba1-and pSTAT5-ir cells were detected and captured with a light microscope (Nikon Eclipse Ni) and a digital 2MP Slider CCD camera (Diagnostic Instruments, Sterling Heights, MI, USA) using 4–40× objectives and Spot RT3 software. Contrast and sharpness of the images were adjusted using the “levels” and “sharpness” commands in Adobe Photoshop CS 8.0.

Mouse and rat brain atlases^73^^,^^74^ were used to identify brain areas in mice and rats, respectively. The same anatomical locations were analyzed in each animal. The density of labeled neurons was expressed as cell number/mm^2^. The number of labeled cells in the selected brain area was counted using ImageJ software, version 1.50i^67^ in 5 photomicrographs taken per region of interest across at least 3 serial sections.

Statistical analysis was performed using 2-way ANOVA test with the type of treatment (PLX3397 vs. control) being one parameter and the brain regions being the repeated parameter in case of Iba1 labeling. For pSTAT5 labeling, the statistical analysis to compare the density of cells in the mediobasal hypothalamus between control and PLX3397 treated groups was performed with unpaired t-test.

#### Monitoring the weight gain of pups

The weight gain of pups was monitored during the treatments and also in the test periods. The weight of pups was measured individually in the Exp. 1 and 2. In Exp. 3, the weight of the litter was measured. During treatments, the weight of pups was measured weekly on the first 2 weeks, and daily on the last week.

In Exp. 2, the weight of pups stopped growing on treatment day 15.50 ± 0.22. Therefore, mothers were given 3 foster pups (3-5 days old) to maintain lactation and maternal care until the end of the experiment. At the start of the test period (21^st^ postpartum day of the mothers), all of the mothers received 3 new, 2-3 daysold foster pups. The weight of these foster pups was measured every day thereafter during the experiment.

In Exp. 3, mothers received foster pups (10 pups per mother) on the 17^th^ postpartum day to maintain their lactation and maternal behavior. At the start of the test period (23^rd^ postpartum day of the mothers), each mother received 10 new, 2-3 daysold foster pups. The weight of these foster pups was measured every day thereafter during the experiment.

The statistical analysis of weight gain was performed with two-way ANOVA with the treatment being the independent parameter and the body weights on consecutive days the repeated parameter. The multiple comparison with Sidak’s test was performed only in Exp. 3 as data were missing in the other experiments since some pups were taken out due to their weight loss.

#### Determination of weight gain by pups during nursing bouts

The weight of pups was measured in the morning at 8.00 AM on the 3^rd^ test day (3^rd^ postpartum day in Exp. 1 but 24^th^ postpartum day in Exp. 2 and 3) in each experiment. After that, the pups were isolated from the mothers for 4 hours as described previously.^12^^,^^19^ During the isolation, the pups were placed together in a cage, which contained a heating pad to prevent the pups from cooling down. All pups survived this separation period. Before the pups were returned to the mothers, their weight was measured. The pups and the mothers were left together for 1 h, during which suckling took place. At the end, the weight of pups was measured again. The statistical analysis on the weight changes of the pups was carried out with paired t-test.

#### Measurement of suckling-induced prolactin release

##### Implantation of osmotic minipumps

Osmotic minipumps for continuous 28 days injection (ALZET Micro-Osmotic Pump model 2004, Durect™, Cupertino, CA, USA), loaded with PLX3397 (0.5 mg/ml) or artificial cerebrospinal fluid (ACSF; 147 mM NaCl, 3.5 mM KCl, 2 mM CaCl2, 1 mM MgCl2, pH = 7.2) as vehicle control were used for intracerebroventricular (icv.) injection via cannulae (ALZET Brain Infusion Kit 2, Durect™) in rat mothers (n = 10+10). Icv. cannulae and osmotic minipumps were applied on the 2^nd^ postpartum day. For that, rats were anesthetized with an intramuscular injection of anesthetic mix containing 0.3 ml/300 g body weight ketamine (67 mg/kg) and 0.2 ml/300 g body weight xylazine (13 mg/kg). Then, rats were placed in a stereotaxic apparatus. The skin was cut and a hole of about 1 mm in diameter was drilled in the left side of the skull above the lateral ventricle at the following coordinates: −0.5 mm anteroposterior to the bregma; 1.4 mm lateral to the bregma; 3.6 mm ventral to the surface of the brain. Cannula were inserted into the lateral ventricle and secured to the skull with cranial plastic cement. The pumps were placed subcutaneously at the back of the animals. After surgery, Tardomyocel® comp. Antibiotics III (0.1 ml/kg) was administered to the animals subcutaneously for 3 days after the surgery to prevent infection.

##### Implantation of jugular cannula

Mother rats received 25-mm-long sterile polyethylene jugular catheters (Plastics One) under 0.3 ml/300 g body weight ketamine (67 mg/kg) and 0.2 ml/300 g body weight xylazine (13 mg/kg) anesthesia one day before blood sampling began. The right common jugular vein was opened, a catheter was placed in the vessel, sutured, and pulled through the skin between the scapulae. After the insertion of the catheter, it was filled with heparinized saline and closed with metal pins.^75^

##### Blood sampling

One day after the implantation of the jugular cannula, blood sampling started for the measurement of serum prolactin level. The first blood sample was taken before removing the pups for 4 h from the mothers. After a 4 h separation of the pups, a second blood sample was taken 5 min before the pups were returned to their mothers. After the mother and the pups were reunited, suckling usually started immediately and never more than 5 min after the pups were given back to the mothers. Blood samples were taken at 5, 15, 30, and 60 min after pups were returned to their mothers. At each time point, 200 μl blood was collected and the same amount of heparinized saline was injected back into the circulation. Blood was centrifuged at 4°C for 10minat 12.000 g to separate plasma, which was then stored at -20°C until measurement of prolactin concentration. Immediately after the last blood sampling, the animals were transcardially perfused for Iba1 and pSTAT5 immunohistochemistry.

##### Prolactin assay

Serum prolactin level was measured with radioimmunoassay in triplicates, which was processed as described previously.^57^

The statistical analysis was carried out with 2-way ANOVA followed by Sidak’s multiple comparison test with the type of treatment (PLX3397 vs. control) being one parameter and time points of prolactin level measurements being the repeated parameter (n = 10 treated and 9 in control mothers).

#### Maternal behavioral tests

##### Spontaneous maternal behavior

The number of pups of the mother mice (n = 6 mothers per group) was reduced to 3 immediately after parturition. In the case of rats (n = 10 treated and 9 in control mothers), the number of pups was reduced to 10. Before the treatment, the animals were placed into new cages and kept there individually throughout the experiment. The maternal behavior of the animals was examined on postpartum days 3–9. Spontaneous maternal behavior was tested for 30 min on the 2^nd^ test day (2^nd^ postpartum day in Exp. 1 while 23^rd^ postpartum day in Exp. 2 and 3). Immediately before the start of the test, the dams were placed outside of their home-cage, then their nests were destroyed, and three pups were placed at the farthest corner from the original place of the nest. Return of the adult to the cage marked the beginning of the assay. All observations were recorded in the same room, at the same time of the day, using a camera located laterally to the clear Plexiglass cages. Each test was video recorded using a SJCAM SJ4000 FULLHD action camera. Recorded videos were analyzed by Solomon Coder^68^ software (https://solomon.andraspeter.com/). In spontaneous parental behavior tests, pup-associated (suckling, kyphosis, nest building, and grooming of pups) and non-pup associated behaviors (exploration, self-grooming) were scored. The statistical analysis of maternal behavior was carried out with two-way ANOVA with treatment and behavioral elements being the 2 parameters.

##### Pup retrieval test

Pup retrieval tests were performed on the 1^st^ test day (1^st^ and 22^nd^ postpartum day, respectively) in Exp. 1 and 2 (6 mothers groups; 3 pups per mother), and on the 3^rd^, 6^th^ and 10^th^ test days (postpartum days 24^th^, 27^th^, and 31^st^) in Exp. 3 (n = 10 treated and 9 control mothers; 10 pups per mother) as described previously.^57^ Briefly, the pups were placed in the corner of the cage opposite to the nest. The mothers retrieved the pups back into the nest one by one, the timing of which was registered. The pup retrieval test lasted for 5 min in mice and 10 min in rats. Pup retrieval time was determined as the moment when adults picked up the last pup to carry it to the nest.

In Exp. 1 and 2, the statistical analysis was performed with unpaired t test using the Welch’correction because the F test indicated significant differences between the variances of the control and treated groups. In Exp. 3, the statistical analysis was performed using 2-way Repeated Measures ANOVA test with the drug vs. control treatment being one parameter, and the days being the other, repeated parameter in Exp. 2.

#### Testing of anxiety- and depression-like behaviors

The open field test was performed on the 5^th^ test day (5^th^ postpartum day) in Exp. 1 (6 mothers per group) and 2 (26^th^ postpartum day;6 mothers per group). The tests lasted for 10 min, during which videorecording was performed. The open field box was made from polypropylene (40 x 40 x 60 cm). After each experiment, the open field arena was cleaned with 70% ethanol and then with dry wipe. The videos were evaluated for stretching, grooming, freezing, running, sniffing, walking and jumping behaviors and the regions of box: center, wall and corner. For comparison of control and treated groups, we used unpaired Student’s t-test.

To assess anxiety level, mothers were tested in the elevated plus maze (EPM) for 10 min on the 5^th^ test day (5^th^ postpartum day in Exp. 1, 26^th^ postpartum day in Exp. 2 and 3) in each experiment (n = 6 in Exp. 1 and 2; n = 10 treated and 9 control in Exp. 3). The test started after 1 h of resting time for the animals after the open field test. The apparatus consisted of two open arms (OA) (mice: 25x5; rats: 40x6 cm) and two similar size closed arms (CA) connected by a central platform (mice: 5x5, rats: 6x6 cm). The apparatus was elevated to 40 cm above the floor. At the beginning of the session, each mouse/rat was placed into the center of the maze, facing one of the OAs and behavior was video recorded for 10 min. We used a video camera supported by a tripod. Each test was video recorded using SJCAM SJ4000 FULLHD action camera. The number of entries and the time spent in each arm of the maze were analyzed with Salomon Coder program or the SMART Video Tracking software (Panlab Harvard Apparatus, USA). After each EPM test, the animals were immediately returned to their home cages, and the maze was cleaned with 70% ethanol solution to remove odor trails.

On the 5^th^ test day (5^th^ postpartum day in Exp. 1, 26^th^ postpartum day in Exp. 2 and 3) in each experiment (n = 6 per group in Exp. 1 and 2; n = 10 treated and n=9 control in Exp. 3), the last test of the animals was the forced swim test (FST). The test starts after 1 hour of resting time for the animals after the EPM test. Plastic cylindrical tanks (mice: 30 cm height x 20 cm diameters; rats: 50 cm height x 25 cm diameters) were used. The temperature of the water was 24 °C. The test lasted for 6 min in each case. After the FST test, animals were wiped with a towel and returned to their home cages. The tanks were cleaned with 70% ethanol solution to remove odor trails. The videos were evaluated for swimming, climbing and floating behaviors. For comparison of control and treated groups, we used unpaired Student’s t-test.

#### Injection of PLX3397 to the pups

Intraperitoneal injection of PLX3397 (100 mg/kg) was injected into the pups intraperitoneally because this is the estimated concentration of the drug in the mothers, too. The weight of the pups was measured right before injection and a day later. On that day, the effect of PLX3397 on suckling by the pups was also measured. The pups were removed from the mothers for 4 hours when they received another dose of PLX3397 (100 mg/kg) 1 hour before returning to their mothers. The body weight of pups was measured immediately before the PLX3397 injection, and also at the end of the 1-h-long suckling bout.

### Quantification and statistical analysis

All statistical tests, significance levels and samples sizes are reported in the corresponding paragraph of the STAR Methods and results sections and the figure legends. Sample size (*n)* represents the number of mothers, pups or litter per group, which is specified in each case. All statistical calculations were carried out using GraphPad Prism (GraphPad Software, LLC, released 2020, version 9.0.0.). Data were first tested with Shapiro-Wilk test for normality. If the data were normally distributed, we used two-tailed paired t-test for two groups, which were related to each other (e. g. apparent weight gain during suckling). When the comparison was made between two different groups, we used two-tailed unpaired t-test (e. g. comparison of the density of pSTAT5 labeled neurons in the mediobasal hypothalamus of control and treated mothers). The pup retrieval times in Exp. 1 and 2 showed different variances between the 2 groups in F test. Therefore, the Welch’s correction of the unpaired t-test was used. In case of the comparison between more than 2 normally distributed groups, two-way ANOVA (e. g. comparing different maternal behavioral elements between the control and the treated groups) or repeated measures ANOVA (e. g. measuring body weight and pup retrieval times at consecutive days or prolactin levels at different time points) were performed followed by Sidak’s multiple comparisons tests. We list the p value in all cases. Statistical analyses were considered significant for p< 0.050 indicated as follows: 0.010 < ∗p< 0.050, 0.001 < ∗∗p< 0.010, ∗∗∗p< 0.001. All values are expressed as the mean ± standard error of mean (SEM).

## Data Availability

• Single-cell RNA-seq data and Western blot images have not been generated in the present study. Microscopy data reported in this paper will be shared by the lead contact upon request. • No original code was used in the analysis of the data. • Any additional information required to reanalyze the data reported in this paper is available from the lead contact upon request. • The data presented in this study are available upon request from the lead author.
