# Supplementary material for: Microglia depletion prevents lactation by inhibition of prolactin secretion
Source: iScience. 2023 Feb 24;26(3):106264. doi: 10.1016/j.isci.2023.106264 (PMC10014264; doi:10.1016/j.isci.2023.106264)
Supplement: Document S1. Figures S1–S3 and Tables S1–S6 [file mmc1.pdf]

iScience, Volume *26*

Supplemental information

Microglia depletion prevents lactation by inhibition of prolactin secretion

Vivien Csikós, Szilvia Oláh, Fanni Dóra, Nikolett Arrasz, Melinda Cservenák, and Arpád Dobolyi

Supplementary figures and tables

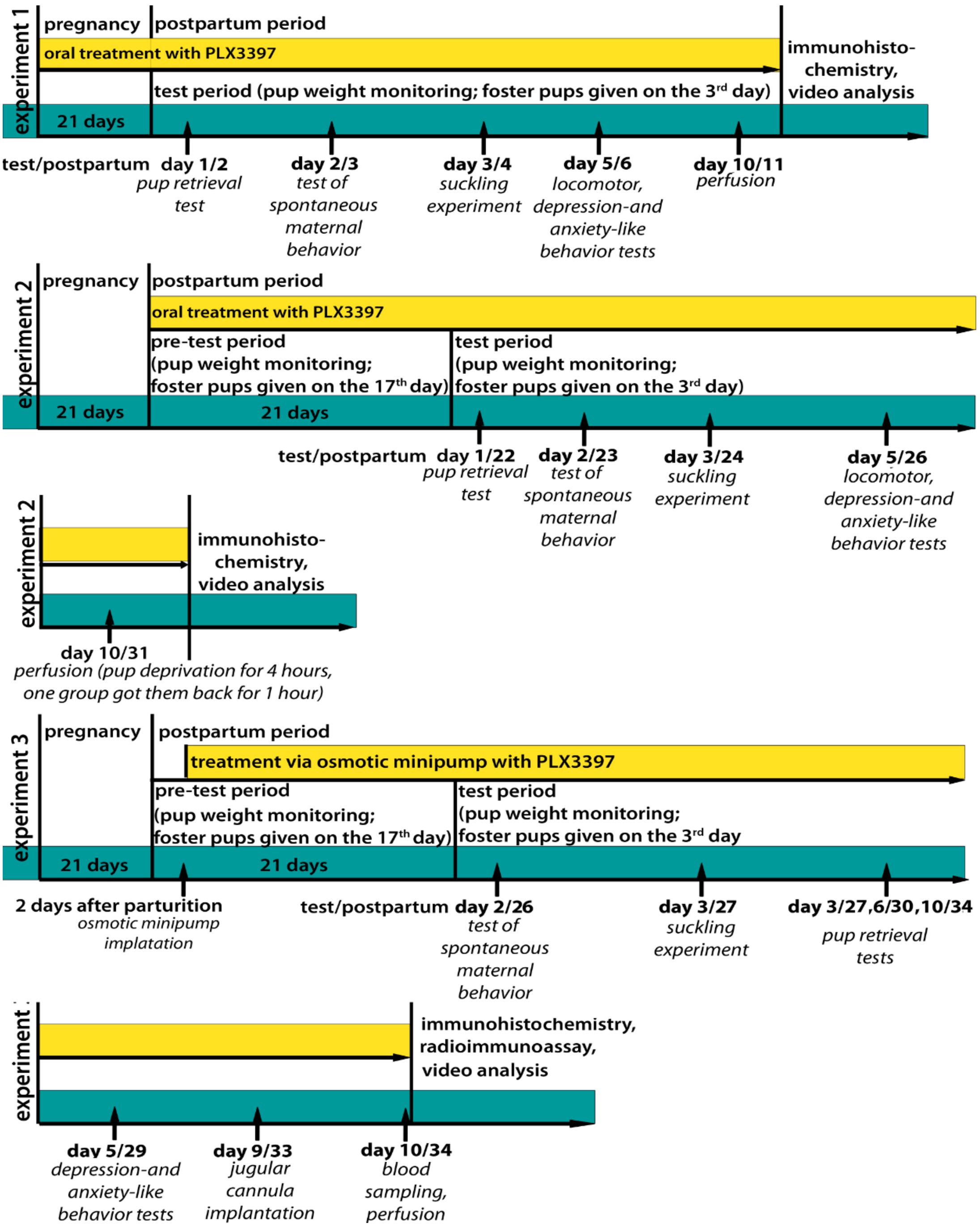

Figure S1. The timeline of each experiment, Related to Figures 1, 2 and 3.

The timeline shows the lengths of the 3 experiments and the timing of the tests and procedures performed on the PLX3397 treated animals and the vehicle treated control animals. The test period always started 21 days after the beginning of treatment to leave sufficient time for the drug to exert its action on the microglia. At the end of the test period, the animals were perfused for immunohistochemistry, the radioimmunoassay measurements and videoanalysis of the behaviors of the animals were evaluated.

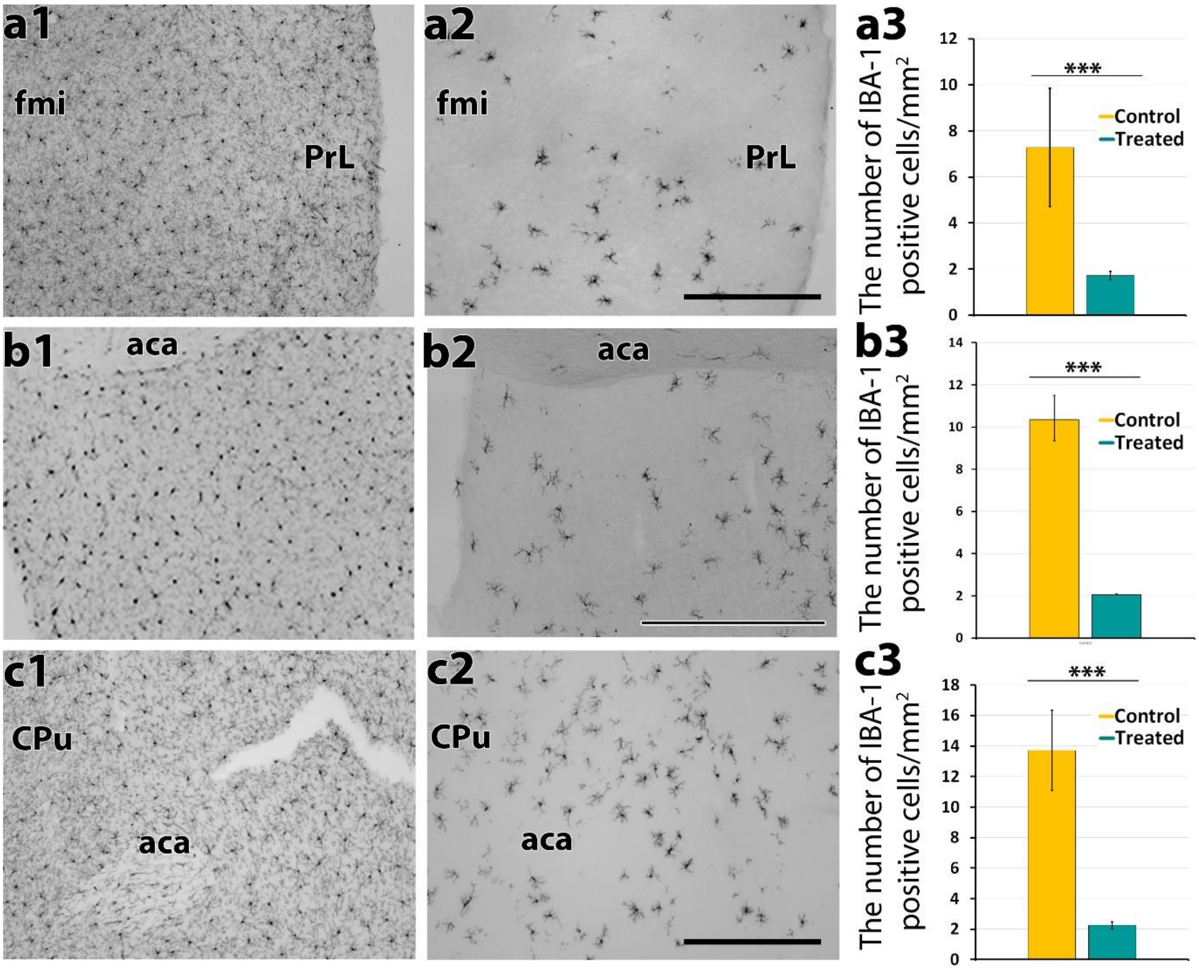

Figure S2. Brain regions with microglial cells labeled with Iba1 in response to PLX3397 treatment in Exp. 1., Related to Figure 1.

a1, Iba1 positive cells are shown in the medial prefrontal cortex (mPFC) in control animals. **a2,** A sample from a PLX3397 treated animal. **a3,** The number of Iba1 labeled cells in 1 mm^2 in the mPFC (n = 6 mothers per group). **b1,** Iba1 positive cells in the medial preoptic area (MPOA) of a control animal. **b2,** A sample from the MPOA of a treated animal. **b3,** The number of Iba1 positive cells in 1 mm^2 in the MPOA (n = 6 mothers per group). **c1,** A section containing the Accumbens nucleus (NAcc) of a control dam while **c2** shows the treated mother, and **c3** is a diagram about the density of cells in the 2 groups (n = 6 mothers per group). The columns represent the density of Iba1 labeled cells in control animals (yellow) on the left, and the treated group (green) on the right. The scale bars are 400 µm. Further abbreviations: aca: anterior commissure; fmi: forceps minor of the corpus callosum; PrL: prelimbic cortex. ***: $p < 0.001$.

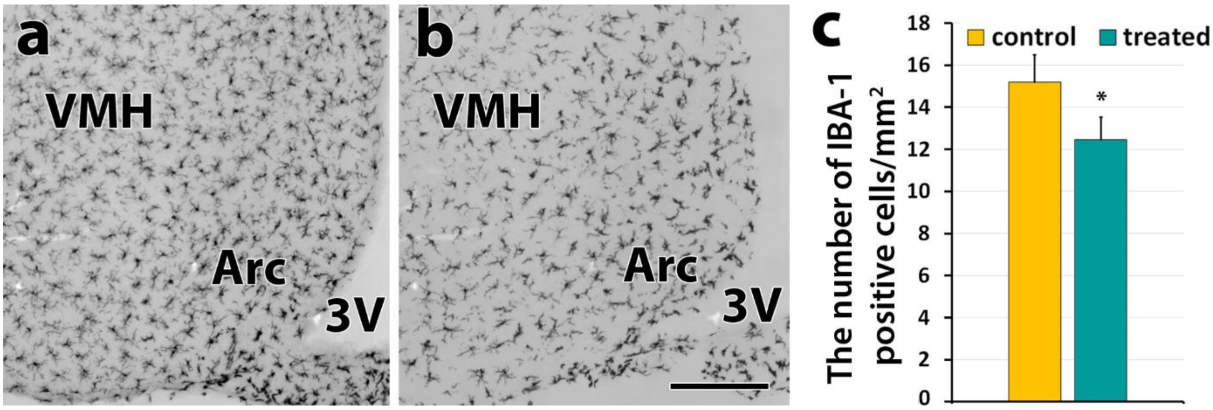

Figure S3. Mediobasal hypothalamus with microglial cells labeled with Iba1 in response to intracerebroventricular PLX3397 treatment in Exp. 3., Related to Figure 3.

a, Iba1 positive cells are shown in the arcuate nucleus (Arc) and the ventromedial hypothalamic nucleus (VMH) in a control animal. **b,** Same brain area as in a panel from a PLX3397 treated animal. **c,** The number of Iba1 labeled cells in 1 mm^2 in the area ($n = 10$ treated and 9 control mothers). The scale bar is 250 µm. Further abbreviations: 3V: third ventricle. *: $p < 0.05$.

Table S1. The density of Iba1 labelled cells in several brain regions in the different experiments, Related to Figures 1, 2, and 3.

The number of cells / mm^2	Experiment 1 *(treatment during pregnancy and in the postpartum)*		Experiment 2 *(treatment only in the postpartum)*		Experiment 3 *(treatment via osmotic minipump only in the postpartum)*	
	Control	PLX3397 treated	Control	PLX3397 treated	Control	PLX3397 treated
**Medial prefrontal cortex (mPFC)**	7.28 ± 2.58	1.72 ± 0.19	7.94 ± 1.71	5.95 ± 0.32	15.05 ± 0.80	11.90 ± 1.51
**Accumbens nucleus (NAcc)**	13.71 ± 2.62	2.24 ± 0.24	9.27 ± 0.65	4.10 ± 0.37	12.54 ± 1.99	12.06 ± 1.56
**Bed nucleus of the stria terminalis (BNST)**	10.42 ± 2.35	2.27 ± 0.35	5.15 ± 0.21	5.21 ± 1.07	13.67 ± 1.23	11.01 ± 2. 04
**Medial preoptic area (MPOA)**	10.35 ± 1.15	2.05 ± 0.06	9.03 ± 0.28	4.14 ± 0.55	13.13 ± 1.84	11.15 ± 1.85
**Paraventricular nucleus (PVN)**	11.80 ± 1.60	1.91 ± 0.32	3.23 ± 0.23	6.87 ± 0.36	18.63 ± 8.33	13.22 ± 1.04

The table shows the number of Iba1 labeled cells per mm^2 in different brain regions. The difference between the control and treated groups was significant in Exp. 1 and 2. In Exp. 3, the reduction was not significant in the brain regions listed in the table but a tendency for decrease was observed.

Table S2. The weight of pups during the experiments, Related to Figures 1, 2, and 3.

Experiment 1							
Control				PLX3397 treated			
test day 1 (g)	test day 2 (g)	test day 3 (g)	test day 4 (g)	test day 1 (g)	test day 2 (g)	test day 3 (g)	test day 4 (g)
1.29 ± 0.07	1.69 ± 0.10	1.94 ± 0.09	2.12 ± 0.43	1.88 ± 0.04	1.68 ± 0.01	-	-
1.22 ± 0.06	1.68 ± 0.10	1.97 ± 0.10	2.53 ± 0.28	1.66 ± 0.05	-	-	-
1.16 ± 0.01	1.60 ± 0.02	2.01 ± 0.04	2.43 ± 0.07	1.60 ±0.10	1.41 ± 0.03	-	-
1.23 ± 0.10	1..72 ± 0.10	1.47 ± 0.09	1.52 ± 0.10	1.51 ± 0.01	-	-	-
1.29 ± 0.05	1.69 ± 0.08	1.79 ± 0.10	2.04 ± 0.39	1.76 ± 0.02	1.60 ± 0.10	-	-
1.86 ± 0.05	2.13 ± 0.07	2.53 ± 0.04	2.90 ± 0.08	2.92 ± 0.01	-	-	-

Experiment 2							
Control				PLX3397 treated			
test day 1 (g)	test day 2 (g)	test day 3 (g)	test day 4 (g)	test day 1 (g)	test day 2 (g)	test day 3 (g)	test day 4 (g)
1.51 ± 0.12	1.90 ± 0.12	2.85 ± 0.01	3.72 ± 0.03	2.12 ± 0.21	2.12 ± 0.05	2.10 ± 0.01	-
2.00 ±0.32	2.60 ±0.10	3.26 ± 0.02	4.61 ± 0.01	1.91 ± 0.43	1.90 ± 0.01	1.81 ± 0.02	-
2.56 ± 0.51	3.00 ± 0.21	3.81 ± 0.10	3.84 ± 0.01	1.71 ± 0.02	1.60 ± 0.01	1.63 ± 0.04	-
1.22 ± 0.16	1.92 ± 0.03	2.31 ± 0.10	3.16 ± 0.05	2.87 ± 0.55	-	-	-
1.26 ± 0.13	1.39 ± 0.09	1.54 ± 0.05	2.64 ± 0.10	1.88 ± 0.17	1.87 ± 0.12	-	-
1.42 ± 0.12	1.60 ± 0.04	1.70 ± 0.01	2.05 ± 0.13	1.64 ± 0.01	1.61 ± 0.04	-	-

Experiment 3															
Control								PLX3397 treated							
test	8.22 ± 0.02	test	9.68 ± 0.02	test	11.0 ± 0.02	test	12.9 ± 0.02	test	8.09 ± 0.01	test	9.31 ± 0.01	test	10.2 ± 0.04	test	11.8 ± 0.05

	7.71 ± 0.01	9.09 ± 0.02	10.3 ± 0.02	12.0 ± 0.01	8.43 ± 0.04	9.00 ± 0.05	10.6 ± 0.07	11.7 ± 0.07
	5.27 ± 0.02	6.26 ± 0.02	7.35 ± 0.02	8.27 ± 0.02	5.38 ± 0.10	6.26 ± 0.09	7.23 ± 0.08	8.21 ± 0.09
	5.20 ± 0.12	6.26 ± 0.12	7.38 ± 0.10	8.54 ± 0.11	5.58 ± 0.01	6.56 ± 0.07	6.59 ± 0.08	7.62 ± 0.10
	5.32 ± 0.04	6.14 ± 0.09	7.08 ± 0.10	7.96 ± 0.07	5.20 ± 0.01	6.10 ± 0.02	7.32 ± 0.01	8.21 ± 0.01
	92.6 ± 0.01	9.06 ± 0.02	12.5 ± 0.02	14.2 ± 0.03	9.29 ± 0.01	9.74 ± 0.05	9.47 ± 0.01	10.8 ± 0.01
	6.81 ± 0.01	7.65 ± 0.01	8.48 ± 0.03	9.41 ± 0.06	12.3 ± 0.12	14.2 ± 0.10	15.2 ± 0.08	17.8 ± 0.10
	14.4 ± 0.12	16.7 ± 0.10	18.8 ± 0.08	21.40 ± 0.12	14.2 ± 0.11	15.2 ± 0.10	16.9 ± 0.10	19.4 ± 0.09
	9.15 ± 0.04	10.7 ± 0.02	13.9 ± 0.02	15.0 ± 0.03	18.2 ± 0.01	20.2 ± 0.02	22.5 ± 0.01	24.9 ± 0.01

The table summarized the weight gain of pups / foster pups in each experiment. The weight of pups is averaged per mothers. In Exp. 2, mice mothers received new foster pups on the 21st postpartum day. The measurements started 2 days later on the 23rd postpartum day (1st test day). So the test days 1, 2, 3, and 4 in the table correspond to the mother's postpartum days 23, 24, 25, and 26, respectively. In Exp. 3, rat mothers received new the foster pups on the 23th postpartum day, which was considered as test day 1. The measurements started 2 days later on the 25th postpartum day (1st test day). So, the test days 1, 2, 3, and 4 in the table correspond to the mother's postpartum days 25, 26, 27, and 28, respectively.

Table S3. The summary table of foster pups used in the study, Related to figures 1, 2, and 3.

	**Experiment 1**		**Experiment 2**		**Experiment 3**	
	**Control**	**PLX3397 treated**	**Control**	**PLX3397 treated**	**Control**	**PLX3397 treated**
**Did mothers get foster pups?**	×	✓	✓	✓	✓	✓
**The time point when mothers received foster pups**	-	on the 2nd day after parturition (2nd test day) and in some case if it was needed, on the 5th test day (5th day after parturition)	on the 21th day after parturition (1st test day)	on the 21th day after parturition (1st test day) and in some case if it was needed, on the 5th test day (26th day after parturition)	on the 21th day after parturition (1st test day)	on the 21th day after parturition (1st test day)
**The period when (foster) pups were weighted**	3rd -6th day	3rd -6th day	3rd day of the test (23th postpartum day) -6th day	3rd day of the test (23th postpartum day) -6th day	3rd day of the test (25th postpartum day) -6th day	3rd day of the test (25th postpartum day) -6th day

The table contains information on which group of animals received foster pups in the experiments. The table also shows the days of measuring the pups.

Table S4. The weight gains of pups during suckling followed by 4 hours of maternal separation, Related to figures 1, 2, and 3.

**Experiment 1**					
**Control**			**PLX3397 treated**		
**morning weight (g)**	**after 4 hours of starvation (g)**	**after 1 hour suckling (g)**	**morning weight (g)**	**after 4 hours of starvation (g)**	**after 1 hour suckling (g)**
3.58 ± 0.07	3.15 ± 0.16	3.09 ± 0.20	3.91 ± 0.51	3.54 ± 0.64	3.51 ± 0.64
3.64 ± 0.14	3.03 ± 0.05	3.24 ± 0.5	3.53 ± 0.02	3.47 ± 0.02	3.44 ± 0.01
3.34 ± 0.13	2.98 ± 0.03	3.17 ± 0.03	4.96 ± 0.07	4.65 ± 0.21	4.58 ± 0.28
3.93 ± 0.03	3.63 ± 0.03	3.81 ± 0.06	6.49 ± 0.01	6.33 ± 0.05	6.12 ± 0.05
2.50 ± 0.01	2.47 ± 0.02	2.51 ± 0.03	5.11 ± 0.06	4.97 ± 0.02	4.93 ± 0.05
3.80 ± 1.17	3.76 ± 1.16	3.79 ± 1.16	2.78 ± 0.05	2.76 ± 0.03	2.59 ± 0.08
**Experiment 2**					
**Control**			**PLX3397 treated**		
**morning weight (g)**	**after 4 hours of starvation (g)**	**after 1 hour suckling (g)**	**morning weight (g)**	**after 4 hours of starvation (g)**	**after 1 hour suckling (g)**
2.00 ±0.32	1.94 ± 0.31	2.05 ± 0.34	1.88 ± 0.17	1.82 ±0.17	1.79 ± 0.16
1.51 ± 0.12	1.46 ± 0.11	1.54 ± 0.11	1.91 ± 0.43	1.80 ± 0.37	1.76 ± 0.34
2.56 ± 0.51	2.47 ± 0.48	2.64 ± 0.54	1.71 ± 0.02	1.63 ± 0.02	1.57 ± 0.05
1.22 ± 0.16	1.10± 0.17	1.33 ± 0.18	2.87 ± 0.55	2.74 ± 0.54	2.62 ± 0.51
1.42 ± 0.12	1.39 ± 0.11	1.45 ± 0.11	2.12 ± 0.21	2.10 ± 0.19	2.10 ± 0.20
1.26 ± 0.13	1.23 ± 0.09	1.30 ± 0.08	1.64 ± 0.01	1.62 ± 0.01	1.61 ± 0.01
**Experiment 3**					
**Control**			**PLX3397 treated**		
**morning weight (g)**	**after 4 hours of starvation (g)**	**after 1 hour suckling (g)**	**morning weight (g)**	**after 4 hours of starvation (g)**	**after 1 hour suckling (g)**

81.15	80.65	83.86	81.11	80.72	84.60
77.51	76.61	89.93	85.25	84.55	82.87
51.75	52.45	54.54	54.20	53.55	54.16
124.75	123.52	126.95	56.65	56.41	58.05
118.46	117.12	120.90	133.49	131.93	132.82
74.56	73.89	75.42	86.98	86.20	86.91
74.32	73.67	74.89	68.66	67.76	67.50
97.71	96.37	100.52	75.55	73.85	72.60
101.01	98.52	99.67	99.96	97.76	100.26
			104.12	103.24	100.64

The table contains the body weight data in all 3 experiments. The weights of pups are shown in three time points (in the morning, after 4 hours of separation from the mother and 1 hour after returning to the mother for suckling) are shown as mean ± s.e.m. for Exp. 1 and 2. In Exp. 3, only the weight of the litters and not individual pups were measured.

Table S5. The results of the maternal behavior tests, Related to figures 1, 2, and 3.

**Maternal behavior expressed as percentage of duration (%)**	**Experiment 1** *(oral treatment during pregnancy and in the postpartum)*		**Experiment 2** *(oral treatment only in the postpartum)*		**Experiment 3** *(treatment via osmotic minipump only in the postpartum)*	
	Control	PLX3397 treated	Control	PLX3397 treated	Control	PLX3397 treated
**Nursing behavior**	48.58 ± 28.59	37.15 ± 16.05	58.44 ± 31.41	47.46 ± 28.12	45.47 ± 12.05	36.32 ± 9.75
**Other maternal behaviors**	21.90 ± 15.26	41.57 ± 23.04	29.52 ± 20.75	28.96 ± 11.40	17.55 ± 3.55	20.28 ± 4.67
**Non-parental behaviors**	50.28 ± 35.35	33.16 ± 35.17	12.04 ± 10.97	23.58 ± 16.89	37.00 ± 6.60	43.60 ± 8.48

The table contains the results of maternal behavioral experiments. Percentage of time spent with suckling, other maternal care behaviors (pup sniffing, nest building, time spent in the nest) and the non-parental behaviors (exploration, digging, freezing, self-grooming) of the dams are shown.

Table S6. Tests of activity, anxiety and depression-like behaviors, Related to figures 1, 2, and 3.

Behavioral elements expressed in the percentage (%) of the total duration of the test			Experiment 1 *(treatment during pregnancy and in the postpartum period)*		Experiment 2 *(treatment only in the postpartum period)*		Experiment 3 *(treatment via osmotic minipump only in the postpartum period)*	
			**Control**	**PLX3397 treated**	**Control**	**PLX3397 treated**	**Control**	**PLX3397 treated**
**Activity, anxiety- and depression-like behavior**	**Open Field**	**corner**	34.72 ± 5.86	33.01 ± 10.53	36.11 ± 5.71	31.64 ± 5.54	-	-
		**next to the wall**	34.12 ± 2.20	37.82 ± 0.85	42.25 ± 3.76	33.90 ± 2.10	-	-
		**center**	31.17 ± 6.01	22.16 ± 11.39	21.64 ± 6.24	34.47 ± 7.34	-	-
		**stretching**	16.56 ± 3.62	14.92 ± 4.11	29.68 ± 4.29	26.26 ± 4.36	-	-
		**grooming**	3.45 ± 1.34	2.70 ± 0.73	7.42 ± 0.73	5.37 ± 1.21	-	-
		**freezing**	4.06 ± 2.26	4.80 ± 2.29	1.73 ± 0.69	-	-	-
		**running**	2.09 ± 0.96	3.64 ± 2.78	28.90 ± 12.63	21.63 ± 1.41	-	-
		**sniffing**	73.28 ± 2.79	73.43 ± 3.13	30.38 ± 5.73	28.64 ± 4.26	-	-
		**walking**	0.33 ± 0.21	-	8.66 ± 2.99	17.09 ± 1.36	-	-
		**jumping behavior**	0.20 ± 0.15	0.51 ± 0.29	0.07 ± 0.04	1.03 ± 0.44	-	-
	**EPM**	**opened arm**	22.29 ± 2.34	23.25 ± 2.64	18.50 ± 2.15	18.05 ± 2.13	31.70 ± 4.60	35.33 ± 3.43
		**closed arm**	77.71 ± 2.34	76.75 ± 2. 641	78.60 ± 0.96	81.95 ± 2.13	68.30 ± 4.60	64.40 ± 3.39

	**FST**	**swimming**	16.58 ±4.18	21.67 ± 5.03	19.23 ± 2.59	35.77 ± 4.64	20.53 ± 3.73	13.65 ± 3.39
		**climbing**	32.58 ± 8.32	26.44 ± 2.71	40.62 ± 5.90	32.65 ± 9.36	33.43 ± 4.05	36.45 ± 3.89
		**floating**	50.83 ± 11.42	51.89 ± 6.85	40.16 ± 6.74	31.58 ± 6.22	46.03 ± 6.64	48.63 ± 5.47

There was no significant difference between the groups in the open-field, elevated plus maze and forced-swim tests. The data are expressed as the percentage of total duration of the test.
